# *Streptomyces rugosispiralis* sp. nov., a Novel Actinobacterium Isolated from Peat Swamp Forest Soil That Produces Ansamycin Derivatives and Nocardamines

**DOI:** 10.3390/antibiotics12091467

**Published:** 2023-09-20

**Authors:** Trinset Weeraphan, Khomsan Supong, Paranee Sripreechasak, Rumpa Jutakanoke, Supalerk Kowinthanaphat, Somboon Tanasupawat, Pattama Pittayakhajonwut, Wongsakorn Phongsopitanun

**Affiliations:** 1 Department of Biochemistry and Microbiology, Faculty of Pharmaceutical Sciences, Chulalongkorn University, Bangkok 10330, Thailand; trinset.w@chula.ac.th (T.W.); supalerk.k@pharm.chula.ac.th (S.K.); somboon.t@chula.ac.th (S.T.); 2 Department of Applied Science and Biotechnology, Faculty of Agro-Industrial Technology, Rajamangala University of Technology Tawan-ok, Chantaburi 22210, Thailand; 3 Office of Educational Affairs, Faculty of Science, Burapha University, Chonburi 20131, Thailand; paranees@go.buu.ac.th; 4 Department of Microbiology and Parasitology, Faculty of Medical Science, Naresuan University, Phitsanulok 65000, Thailand; rumpaj@nu.ac.th; 5 National Center for Genetic Engineering and Biotechnology (BIOTEC), National Science and Technology Development Agency (NSTDA), Thailand Science Park, Pathum Thani 12120, Thailand; pattamap@biotec.or.th; 6 Natural Products and Nanoparticles Research Units (NP2), Chulalongkorn University, Bangkok 10330, Thailand

**Keywords:** actinomycetota, *Streptomyces*, polyphasic taxonomy, natural products, geldanamycin, nocardamines

## Abstract

Actinomycetes, especially the genus *Streptomyces*, are one of the most promising sources of bioactive natural products. In this study, a novel *Streptomyces* strain, RCU-064^T^, was isolated from a soil sample collected from a peat swamp forest in Thailand. Strain RCU-064^T^ showed the highest 16S rRNA gene sequence similarity (99.06%) with *Streptomyces malaysiensis* NBRC 16446^T^. Based on a polyphasic approach, strain RCU-064^T^ represents a novel species of the genus *Streptomyces*, for which the name *Streptomyces rugosispiralis* sp. nov. is proposed. The chemical isolation of the crude ethyl acetate extracts of the strain led to the isolation of six compounds: (**1**) geldanamycin, (**2**) 17-*O*-demethylgeldanamycin, (**3**) reblastatin, (**4**) 17-demethoxyreblastatin, (**5**) nocardamine, and (**6**) dehydroxynocardamine. These compounds were evaluated for their biological activities. All compounds showed no antimicrobial activity against tested microorganisms used in this study. Compounds (**1**)–(**4**) displayed cytotoxic activity against the NCI-H187 cell line, with IC_50_ values ranging from 0.045–4.250 µg/mL. Cytotoxicity against the MCF-7 cell line was found in compounds (**1**) and (**3**) with IC_50_ values of 3.51 and 1.27 µg/mL, respectively. Compounds (**5**) and (**6**) exhibited cytotoxicity only against Vero cells (IC_50_ of 16.57 µg/mL) and NCI-H187 cells (IC_50_ of 13.96 µg/mL), respectively. These results indicate that peat swamp forest soil remains a promising reservoir of novel actinomycetes capable of producing bioactive natural products.

## 1. Introduction

Bioactive metabolites are mostly produced from various genera of actinomycetes, including members of *Streptomyces*, *Actinomadura*, *Nonomuraea*, *Micromonospora,* and *Verrucosispora* [1]. *Streptomyces* is the most important and dominant genus within the actinomycete groups. This genus belongs to the family *Streptomycetaceae*, which have a high G+C content in their DNA and form extensively branched substrate and aerial mycelia that later differentiate into spore chains [2]. *Streptomyces* can be differentiated from closely related genera by the presence of *LL*-diaminopimelic acid (*LL*-DAP) in the cell-wall peptidoglycan, but without diagnostic sugars in a whole-cell hydrolysate [3]. Streptomycetes are widely distributed in nature and can be found everywhere, including in terrestrial soils and marine samples [4,5]. In addition, some *Streptomyces* strains are associated with plants, lichens, and insects [6,7,8]. Streptomycetes are a major source of bioactive metabolites, with more than 70% of known antibiotics being derived from this genus [1]. Streptomycetes have become very useful in the search for bioactive metabolites because they can produce different chemical core-structures, such as macrolides, polyketides, terpenes, and non-ribosomal peptides [9].

Cancer is one of the leading causes of death. According to the Global Cancer Statistic 2020, it was estimated that in 2020 there were 19.3 million new cancer cases and 10.0 million deaths from cancer [10]. *Streptomyces* species are a source of small-molecule anticancer compounds. According to a genome mining study, the genomes of streptomycetes harbor a variable distribution of antitumor biosynthetic gene clusters (BGCs) and so these bacteria are a promising source of molecules for antitumor drug discovery [9]. Examples of anticancer drugs derived from streptomycetes are actinomycin, bleomycin, and doxorubicin [11].

Peat swamp forests are found mainly in Southeast Asia. These ecosystems represent a unique character, distinct from other soil habitats, in terms of their acidic and waterlogged conditions [12]. Peat swap forest soils harbor a large amount soil microbes and have been recognized as a promising source of new actinobacteria. In the past few decades, several novel actinobacteria, including *Dactylosporangium sucinum*, *Streptomyces actinomycinicus*, and *Nocardia rayongensis*, have been isolated from these ecosystems [13,14,15]. In this present study, we describe a taxonomic study of the new *Streptomyces* strain RCU-064^T^ based on a polyphasic approach. The bioactive metabolites produced by the strain were elucidated using column chromatography, including Sephadex LH-20 column and high-performance liquid chromatography (HPLC) for purification of the compound, and nuclear magnetic resonance (NMR) and mass spectral analyses for structure determination. The isolated compounds were analyzed for their (i) cytotoxicity against cell lines derived from oral human epidermoid carcinoma (KB), human breast cancer (MCF-7), and human small cell lung cancer (NCI-H187), (ii) anti-malarial activity against the multidrug-resistant *Plasmodium falciparum* K-1, and (iii) anti-bacterial and anti-fungal activities.

## 2. Results

### 2.1. Genomic Feature and Phylogeny

The genome of strain RCU-064^T^ has been deposited at GenBank under the accession number JANIAA000000000, and is 11,017,810 bp in size with a G+C content of 71.3%. The extracted 16S rRNA gene sequence from the genome was 1524 bp.

**Figure 1:**
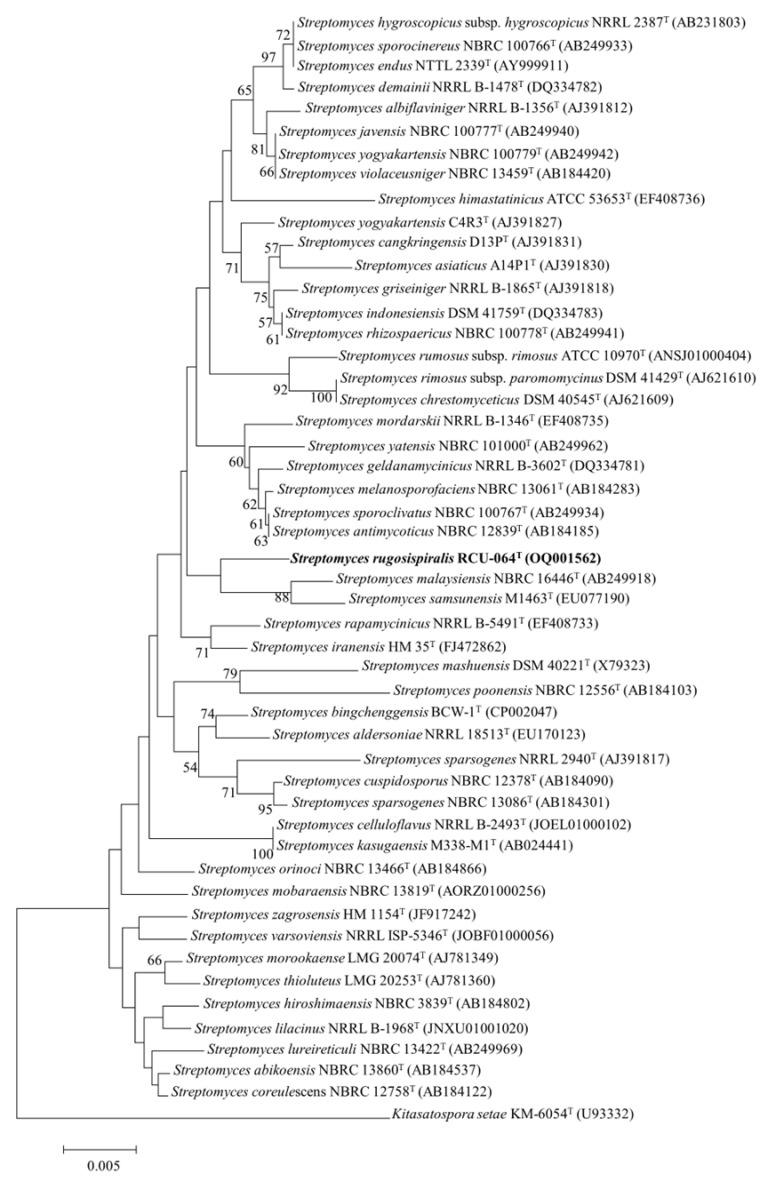
A ML phylogeny based on the 16S rRNA gene sequences of strain RCU-064^T^ and its closely related *Streptomyces* species. *Kitasatospora setae* KM6054^T^ was used as an out group. The number at the branch node indicates the bootstrap value (% from 1000 replicates). Only bootstrap values above 50 are shown. Bar, 0.005 substitutions per nucleotides. The bold text shows the position of *Streptomyces rugosispiralis* RCU-064^T^ in this phylogenetic tree.

**Figure 2:**
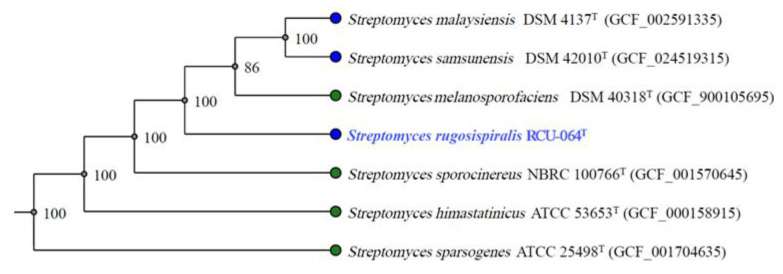
Core-gene ML phylogeny based on the genome of strain RCU-064^T^ and the closely related *Streptomyces* species. The number on the branch nodes indicate the bootstrap values (% from 1000 replicates). The blue text shows the position of *Streptomyces rugosispiralis* RCU-064^T^ in this phylogenetic tree.

Strain RCU-064^T^ showed the highest 16S rRNA gene sequence (accession number: OQ001562) similarity value of 99.06% with *Streptomyces malaysiensis* NBRC 16446^T^. The maximum likelihood (ML) phylogeny based on 16S rRNA gene sequences showed that strain RCU-064^T^ positioned in the same node with *S. malaysiensis* NBRC 16446^T^ and *S. samsunensis* M1463^T^ (Figure 1). The core-gene ML phylogeny exhibited that strain RCU-064^T^ positioned in the node with *S. malaysiensis* NBRC 16446^T^, *S. samsunensis* M1463^T^, and *S. melanosporafaciens* DSM 40318^T^ (Figure 2).

The computed ANIb and ANIm values between RCU-067^T^ and its closely related type strains range from 89.3% to 90.2% and 92.1% to 91.9%, respectively. Furthermore, the dDDH values between strain RCU-064^T^ and the closely related type strains fall within the range of 43.2% to 43.8% (Table 1). These ANI and dDDH values are lower than the established thresholds of 95–96% for ANI and 70% for dDDH, which are typically employed to distinguish the species [16]. Therefore, it could be concluded that strain RCU-067^T^ is the novel species of the genus *Streptomyces*.

According to the prediction made by the antiSMASH webservice for bacterial BGCs, the genome of strain RCU-064^T^ contains several BGCs, which encompass non-ribosomal peptide synthases, type 1 polyketide synthases (T1PKS), type 2 polyketide synthases (T2PKS), type 3 polyketide synthases (T3PKS), terpene, siderophore, and aryl polyene as showed in Table 2.

### 2.2. Chemotaxonomy and Phenotypic Properties of Strain RCU-064^T^

The cell wall peptidoglycans of strain RCU-064^T^ contained LL-DAP and the *N*-acyl type of muramic acid was acetyl. No diagnostic sugars were detected in whole-cell hydrolysates of strain RCU-064^T^, with only glucose and ribose being detected. The polar lipids were diphosphatidylglycerol, phosphatidylethanolamine, hydroxy-phosphatidylethanolamine, phosphatidylglycerol, phosphatidylinositol, an unidentified phospholipid, two unidentified glycolipids, and five unidentified lipids. Menaquinones were MK-9(H_2_) (10.8%), MK-9(H_4_) (24.7%), MK-9(H_6_) (54.1%), and MK-9(H_8_) (10.4%). The major cellular fatty acids (>4%) were iso-C_16:0_ (21.4%), C_16:0_ (14.4%), anteiso-C_15:0_ (13.0%), iso-C_14:0_ (8.5%), iso-C_15:0_ (8.6%), C_17:0_ cyclo (5.1%), and anteiso-C_17:0_ (4.1%) (Table 3). These chemical composition profiles of strain RCU-064^T^ exhibited the same pattern as other members of the genus *Streptomyces*.

**Figure 3:**
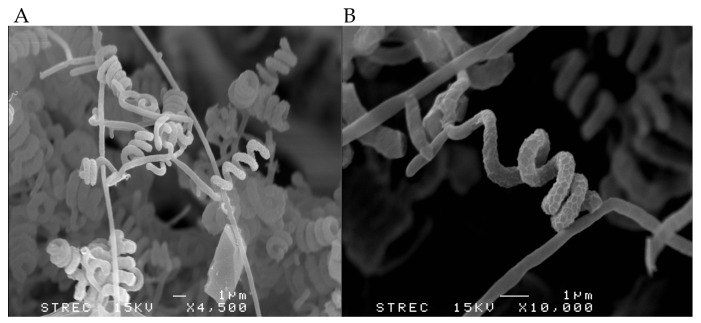
Representative scanning electron micrographs showing the (**A**) spiral spore chains with (**B**) a rugose surface produced by strain RCU-064^T^ when grown on ISP2 medium for 14 days at 30 °C.

Strain RCU-064^T^ grew well on all tested ISP media. White to grey aerial masses could be observed when grown on ISP2, ISP3, ISP4, ISP5, ISP7, and nutrient agar, but aerial masses were absent on ISP6 (Table 4). The strain did not produce soluble pigment on any agar media used in this study. It produced an extensively branched substrate and aerial mycelia. The mature spore chain was of a spiral type and could be observed on the aerial mycelia (Figure 3A) and the surface of the spores is rugose (Figure 3B). The strain exhibited a positive result for starch hydrolysis but negative results for liquefaction of gelatin, milk peptonization, and nitrate reduction. Growth could be observed at 25–37 °C and at pH 5–9 with an optimum pH of 6–7. No growth was observed at 45 °C. The strain tolerated up to 6% (*w*/*v*) NaCl. The biochemical properties could be used to differentiate strain RCU-064^T^ from its closely related *Streptomyces* species (Table 5). The details of the biochemical properties and physiology of strain RCU-064^T^ are summarized in the description of the species.

### 2.3. Description of Streptomyces rugosispiralis sp. nov.

*Streptomyces rugospiralis* (ru.go.si.spi.ra’lis. L. masc. adj. *rugosus*, wrinkled; N.L. masc. adj. *spiralis,* coiled; N.L. masc. adj. *rugosispiralis*, wrinkled and coiled), are Gram-positive, aerobic, mesophilic, filamentous actinomycetes that produce an extensively branched substrate and aerial mycelia. They grow well on ISP2, ISP3, ISP4, ISP5, ISP6, ISP7, and nutrient agar. White to grey aerial masses can be observed when grown on ISP2, ISP3, ISP4, ISP5, ISP7, and nutrient agar, but not on ISP6. The substrate mycelia are dark greenish-yellow to pale greenish-yellow. Type strain does not produce any soluble pigment. Spiral spore chains are produced on the aerial mycelia. The spore surface is rugose. Growth occurs at pH 5–9, with an optimum pH of 6–7, and temperature of 5–37 °C. No growth is observed at 45 °C. Tolerates the presence of NaCl up to 6% (*w*/*v*). Hydrolysis of starch is positive but liquefaction of gelatin, nitrate reduction, and skim milk peptonization are negative. Type strain utilizes arabinose, *myo*-inositol, raffinose, cellobiose, galactose, and rhamnose, and weakly utilizes salicin and fructose, as a sole carbon source but does not utilize ribose and sucrose.

Enzymatic activities for alkaline phosphatase, leucine arylamidase, leucine arylamidase, α-chymotrypsin, acid phosphatase, napthol-AS-BI-phosphohydrolase, and *N*-acetyl-β-glucosaminidase are positive. Weakly positive for esterase (C4), esterase lipase (C8), cystine arylamidase, trypsin, napthol-AS-BI-phosphohydrolase, and α-glucosidase. Type strain shows negative enzyme activities for lipase (C14), α-galactosidase, and α-mannosidase.

Cell wall contains LL-DAP. *N*-acyl type of muramic acid is acetyl. The polar lipids are diphosphatidylglycerol, phosphatidylethanolamine, hydroxy-phosphatidylethanolamine, phosphatidylglycerol, phosphatidylinositol, an unidentified phospholipid, two unidentified glycolipids, and five unidentified lipids. Menaquinones are MK-9(H_2_), MK-9(H_4_), MK-9(H_6_), and MK-9(H_8_). The major cellular fatty acids are iso-C_16:0_, C_16:0_, anteiso-C_15:0_, iso-C_14:0_, iso-C_15:0_, C_17:0_ cyclo, and anteiso-C_17:0_. Type strain RCU-064^T^ (=TBRC 16203^T^, = NBRC 115861^T^) was isolated from soil collected from Nong Jun Rung peat swamp forest, Rayong province, Thailand. The in silico DNA G+C content of the type strain is 71.3% mol%.

### 2.4. Isolation and Structure Elucidation of Metabolites

**Figure 4:**
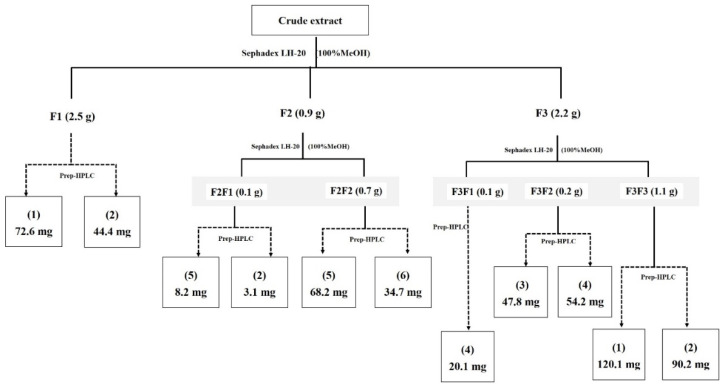
Isolation and purification procedure of compounds from crude EtOAc extract.

**Figure 5:**
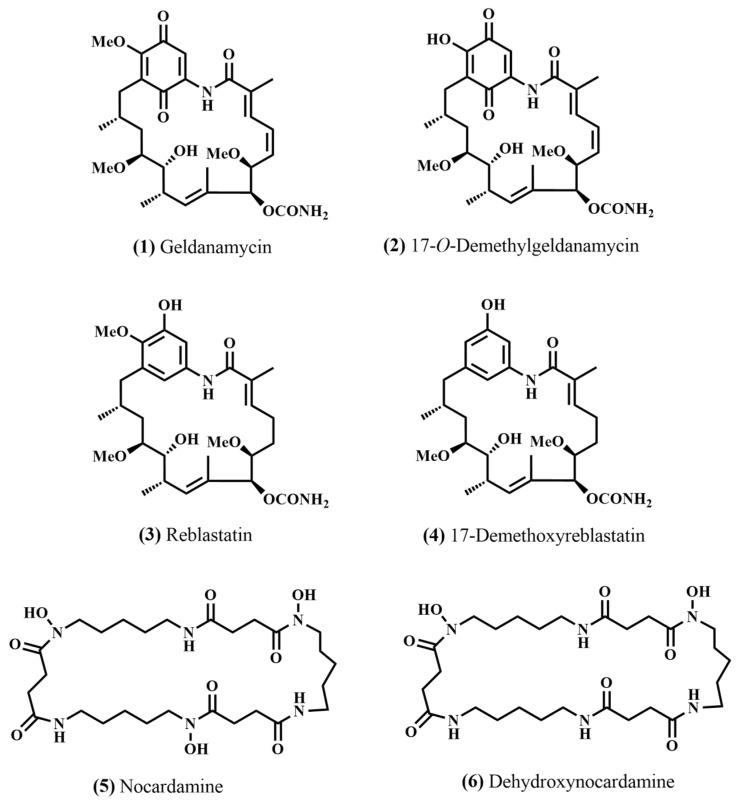
Chemical structure of the isolated compounds (**1**) geldanamycin, (**2**) 17-*O*-demethylgeldanamycin, (**3**) reblastatin, (**4**) 17-demethoxyreblastatin, (**5**) nocardamine, and (**6**) dehydroxynocardamine.

The crude extract of strain RCU-064^T^ was isolated and enriched by Sephadex-LH20, and HPLC column chromatographic techniques to give six compounds (Figure 4). The chemical structure of compounds (**1**)–(**6**) are shown in Figure 5.

- Geldanamycin (**1**): Yellow solid; ^1^H-NMR (500 MHz, CDCl_3_) *δ*: 0.99 (3H, d *J* = 6.55), 1.00 (3H, d *J* = 6.40), 1.75 (1H, m), 1.76 (2H, m), 1.79 (3H, s), 2.02 (3H, s), 2.47 (2H, m), 2.77 (1H, m), 3.29 (3H, s), 3.35 (3H, s), 3.39 (1H, m), 4.12 (3H, s), 3.50 (1H, m), 4.31 (1H, d *J* = 9.22), 5.18 (1H, d *J* = 4.24), 5.81 (1H, d *J* = 9.30), 5.86 (1H, m), 6.57 (1H, m), 6.94 (1H, m), 7.41 (1H, s), and 8.7 (1H, s); ^13^C-NMR (125 MHz, CDCl_3_) *δ*: 12.4, 12.5, 12.3, 22.9, 28.0, 32.2, 32.7, 34.7, 56.7, 57.3, 61.7, 72.7, 81.0, 81.3, 81.4, 108.2, 111.8, 126.2, 127.6, 133.1, 133.3, 134.8, 138.1, 140.5, 153.1, 156.0, 157.0, 184.1, and 185.0. HRESIMS *m/z* 559.2673 [M−H]^−^ (calcd. for C_29_H_39_N_2_O_9_, 559.2661). The ^1^H-NMR, ^13^C-NMR and mass spectra of compound (**1**) are shown in Figures S1, S2 and Figure S13, respectively.
- 17-*O*-Demethylgeldanamycin (**2**): Yellow solid; ^1^H-NMR (400 MHz, CDCl_3_) *δ*: 0.94 (3H, d *J* = 6.93), 1.00 (3H, d *J* = 5.36), 1.79 (3H, s), 1.76 (2H, m), 1.7–1.8 (1H, m), 2.03 (3H, s), 2.44 (2H, m), 2.79 (1H, m), 3.30 (3H, s), 3.36 (3H, s), 3.37 (1H, m), 3.54 (1H. m), 4.32 (1H, d *J* = 9.24), 5.17 (1H, s), 5.80 (1H, d *J* = 9.21), 5.90 (1H, dd *J* = 10.10), 6.57 (1H, dd *J* = 11.7), 6.97 (1H, d *J* = 11.29), 7.41 (1H, s), and 8.96 (1H, s); ^13^C-NMR (100 MHz, CDCl_3_) *δ*: 12.3, 12.4, 12.8, 23.2, 28.1, 32.3, 32.6, 34.5, 56.7, 57.3, 72.9, 81.1, 81.6, 81.8, 108.2, 117.4, 126.1, 127.8, 133.1, 133.4, 134.6, 137.1, 140.6, 153.2, 156.0, 168.1, 183.1, and 184.3; HRESIMS m/z 545.2514 [M−H]^−^ (calcd. for C_28_H_37_N_2_O_9_, 545.2505). The ^1^H-NMR, ^13^C-NMR and mass spectra of compound (**2**) are shown in Figures S3, S4 and Figure S14, respectively.
- Reblastatin (**3**): White solid; Partial spectroscopic data from the ^1^H-NMR (400 MHz, DMSO-*d*_6_) *δ*: 0.79 (3H, d *J* = 6.28), 0.90 (3H, d *J* = 6.53), 1.43 (3H, s), 1.53 (1H, m), 1.67 (3H, s), 1.75 (1H, m), 2.11–2.20 (2H, m), 2.34 (1H, m), 2.36 (2H, m), 2.56 (2H, m), 3.21 (3H, s), 3.33 (3H, s), 3.62 (3H, s), 3.32 (1H, m), 3.28 (1H, m), 3.01 (1H, m), 4.86 (1H, d *J* = 7.35), 4.29 (1H, d *J* = 4.99), 6.29 (1H, s), 5.29 (1H, d *J* = 9.92), 5.85 (1H, b), 6.86 (1H, s), 9.20 (1H, s), and 9.21 (1H, s); ^13^C-NMR (100 MHz, DMSO-*d*_6_) *δ:* 11.6, 12.8, 15.7, 19.9, 23.5, 29.7, 31.0, 33.5, 34.6, 35.7, 56.3, 58.0, 59.7, 73.9, 79.7, 80.5, 81.1, 107.2, 114.5, 129.7, 132.2, 133.3, 133.5, 134.4, 134.5, 142.4, 149.5, 156.0, and 169.9. HRESIMS *m/z* 571.2987 [M+Na]^+^ (calcd. for C_29_H_44_N_2_NaO_8_, 571.2990). The ^1^H-NMR, ^13^C-NMR and mass spectra of compound (**3**) are shown in Figures S5, S6 and Figure S15, respectively.
- 17-Demethoxyreblastatin (**4**): White solid; ^1^H-NMR (500 MHz, DMSO-*d*_6_) δ: 0.79 (3H, d *J* = 6.30), 0.89 (3H, d *J* = 6.43), 1.35 (3H, s), 1.70 (3H, s), 1.78 (1H, b), 2.03–2.15 (2H, m), 2.95 (2H, m), 3.14 (3H, s), 3.28 (3H, s), 4.35 (1H, d *J* = 4.92), 4.83 (1H, d *J* = 7.44), 5.20 (1H, d *J* = 9.44), 5.66 (1H, s), 6.18 (1H, s), 6.24 (1H, s), 6.57 (b), 9.27 (1H, s), and 9.34 (1H, s); ^13^C-NMR (125 MHz, DMSO-*d*_6_) *δ:* 12.1, 13.7, 17.3, 19.0, 23.6, 30.0, 30.8, 33.0, 34.5, 43.1, 56.7, 58.8, 73.5, 79.7, 80.9, 81.2, 106.3, 113.2, 128.4, 130.3, 131.0, 132.1, 133.5, 134.4, 140.5, 141.4, 156.6, and 157.8; HRESIMS m/z 541.2900 [M+Na]+ (calcd. for C_28_H_42_N_2_NaO_7_, 541.2884). The ^1^H-NMR, ^13^C-NMR and mass spectra of compound (**4**) are shown in Figures S7, S8 and Figure S16, respectively.
- Nocardamine (**5**): White solid; ^1^H-NMR (500 MHz, DMSO-*d*_6_) *δ*: 1.21 (2H, m), 1.36 (2H, m), 1.46 (2H, m), 2.27 (2H, m), 2.57 (2H, m), 3.00 (2H, m), 3.44 (2H, t), 7.75 (1H, s), and 9.62 (1H, s); ^13^C-NMR (125 MHz, DMSO-*d*_6_) *δ*: 23.6, 26.3 27.9, 29.1, 30.4, 38.8, 47.3, 172.0, and 172.5. The ^1^H-NMR, ^13^C-NMR and mass spectra of compound (**5**) are shown in Figures S9, S10 and Figure S17, respectively.
- Dehydroxynocardamine (**6**): White solid. ^1^H-NMR (400 MHz, DMSO-*d*_6_) *δ:* 1.21 (2H, m), 1.35 (2H, bs), 1.48 (2H, bs), 2.28 (2H, s), 2.58 (1H, bs), 2.30 (2H, bs), 7.75 (1H, s), and 9.64 (1H, s); ^13^C-NMR (100 MHz, DMSO-*d*_6_) *δ*: 23.8, 24.1, 26.5, 28.1, 28.2, 29.2, 29.3, 29.4, 30.7, 31.8, 38.9, 39.0, 47.6, 171.9, 172.0, and 172.2; HRESIMS m/z 607.3429 [M+Na]^+^ (calcd for C_27_H_48_N_6_NaO_8_, 607.3426); HRESIMS at *m/z* 623.3379 [M+Na]^+^ (calcd for C_27_H_48_N_6_NaO_9_, 623.3375). The ^1^H-NMR, ^13^C-NMR and mass spectra of compound (**5**) are shown in Figures S11, S12 and Figure S18, respectively.

### 2.5. Biological Activity of the Isolated Compounds

All compounds were assayed for cytotoxicity against the three human cancer derived cell lines (KB, NCI-H187, and NCF-7) and the untransformed monkey Vero cell line. Compounds (**1**)–(**4**) showed a high cytotoxic activity against NCI-H187 with IC_50_ values ranging from 0.045–4.250 µg/mL. Cytotoxic activity against the MCF-7 cell line was found in compounds (**1**) and (**3**) with IC_50_ values of 3.510 and 1.270 µg/mL, respectively. All compounds showed no antimicrobial activity against *M. tuberculosis* and anti-*P. falciparum* at the final concentration of 50 µg/mL. In addition, the isolated compounds showed no detected inhibitory activity against the bacteria *Acinetobacter baumannii*, *Escherichia coli* ATCC 25922, and *Mycobacterium tuberculosis* H37Ra; the fungal plant pathogens *Curvularia lunata*, *Magnaporthe grisea*, and *Alternaria brassicicola*; and the yeast *Candida albicans*. In addition, all compounds had no detected inhibitory activity against the neuraminidase (NA) enzyme. In terms of the biological activities, geldanamycin and its derivatives (**1**)–(**4**) mostly showed a cytotoxic activity, but the nocardamine group (**5**) and (**6**) showed cytotoxicity against only the Vero (IC_50_ of 16.57 µg/mL) and NCI-H187 (IC_50_ of 13.96 µg/mL) cell lines, respectively (Table 6).

## 3. Discussion

Based on the results of this polyphasic approach and genomic evidence, it is clear that strain RCU-064^T^ is a novel species in the genus *Streptomyces*, for which the name *Streptomyces rugosispiralis* sp. nov. is herein proposed. Previous studies have highlighted the presence of novel *Streptomyces* in various ecological niches, including soil environments. For instance, *Streptomyces* have been isolated from diverse soil types worldwide, including agricultural, forest, and grassland soils [19,20,21]. Although actinomycetes have been isolated from soil for a century, novel species are still being reported from this type of habitat [4], including peat swamp forest soils. In the past decade, several novel species have been isolated from peat swamp forest soils in Thailand. For example, *Actinomadura rayongensis, Amycolatopsis acidicola, Dactylosporangium sucinum, Nocardia rayongensis, Nonomuraea rhodomycinica, Streptoyces actinomycinicus, Streptomyces acididurans,* and *Streptomyces humicola* [13,14,15,22,23,24,25]. These studies underline the adaptability of actinobacteria to different soil conditions and their significant contribution to the microbial diversity in terrestrial ecosystems. In addition, this indicates the unique characteristic of peat land harbors a high diversity of novel actinomycetes.

It is important to note that the physicochemical characteristics of peat swamp forest soil are unique due to its specific habitat. Peat swamp soils are typically characterized by a high organic matter content, acidic pH, waterlogged conditions, and low nutrient availability [26]. These distinct conditions pose challenges for actinomycetes isolation, as certain species may have specific growth requirements or preferential adaptation to different environmental parameters.

In terms of the isolation of actinomycetes, there are a number of essential factors to consider. The selection of suitable isolation techniques is essential for capturing a diverse population. Common techniques include serial dilution, plating on selective media supplemented with particular carbon or nitrogen sources, and altering the pH conditions [27]. These selective methods promote the development and isolation of actinomycetes, such as *Streptomyces*, while inhibiting the growth of competing microorganisms.

Actinomycetes are renowned for their remarkable ability to produce a diverse array of secondary metabolites, many of which possess significant bioactive properties. These bioactive compounds have attracted considerable attention in various fields, including pharmaceutical, agricultural, and industrial applications. Among the numerous secondary metabolites produced by actinomycetes, a key precursor in the biosynthesis of several important classes of compounds is 3-amino-5-hydroxybenzoic acid (AHBA).

Importantly, AHBA serves as a starter unit or building block in the assembly of polyketide or nonribosomal peptide backbones, leading to the formation of diverse bioactive compounds [28]. It can be classified into three distinct structural classes. The predominant class comprises ansamycins, wherein AHBA acts as a starter unit for the synthesis of a polyketide chain, leading to the formation of a macrocyclic lactam. Another class includes the mitomycins, which involve the combination of AHBA with an aminosugar component, resulting in the formation of unique tricyclic structures. Lastly, the saliniketals constitute a third class, characterized by being “degraded ansamycins,” although this class has only been observed so far in saliniketal compounds [28].

The ansamycins are a family of polyketides that contain naphthalene/benzene or napthaquinone/benzoquinone rings that are connected at nonadjacent positions by an aliphatic chain. These natural compounds exhibit a variety of biological activities and have clinical applications [29]. Many reports have revealed that they have a wide range of biological activities, including anticancer, antiviral, and antibacterial effects. These compounds are distinguished by the presence of a macrocyclic system that is comprised of an aromatic moiety embedded in an alicycle, which has drawn considerable interest from chemical synthesis and biosynthetic researchers [30].

In this study, two classes of bioactive compounds—ansamycin and cyclic peptides—were isolated from strain RCU-064^T^. Geldanamycin (**1**) is the benzoquinone ansamycin, first isolated from the culture broth of *Sreptomyces hygroscopicus* [31]. It exhibited moderate antimicrobial activity against bacteria and fungi with minimum inhibition concentration (MIC) values ranging from 4 to >100 μg/mL. Geldanamycin has been reported to have in vivo anti-parasitic activity against *Syphacia oblevata* but not against *Plasmodium berghei* [31]. However, in this study, the antimicrobial activity against *Bacillus cereus* and *Mycobacterium tuberculosis* of all compounds was negative (MIC > 50 μg/mL). This difference may be attributed to the variation in the tested microorganisms between these studies.

Geldanamycins (**1**) are potent anticancer and antifungal agents, exhibiting their anticancer activity through the inhibition of heat shock protein 90 (Hsp90), a key chaperone protein involved in the stabilization of cancer cell growth and survival [32,33,34]. By inhibiting the function of Hsp90, geldanamycin (**1**) disrupts multiple signaling pathways involved in cancer progression, leading to cell cycle arrest and apoptosis [35]. Previous work showed that geldanamycin (**1**) inhibited the growth of HPV-18-positive HeLa cells, which are a type of cervical cancer cell line, and also showed antifungal activity against *Setosphaeria turcica* plant pathogens [36].

Geldanamycin (**1**) and its derivatives have been reported to be produced as secondary metabolites in several *Streptomyces* species. In Thailand, *Streptomyces* sp. PC4-3 was isolated from a soil sample collected at Samed Island, Rayong province, Thailand, using starch-casein nitrate agar. The culture broth of *Streptomyces* sp. PC4-3 was extracted with ethyl acetate, concentrated under low pressure to obtain the crude extract, and then subjected to silica gel flash column chromatography and NMR spectroscopic analysis, revealing the active component to be geldanamycin (**1**) [37]. Likewise, the crude extracts of *Streptomyces* sp. BCC71188, which was isolated from a soil sample at Nakhon Si Thammarat Province, Thailand, were subjected to Sephadex LH-20 column chromatography followed by HPLC to yield 19 compounds, including 17-O-demethylgeldanamycin (**2**) [38].

Since geldanamycin (**1**) has demonstrated cytotoxic effects, numerous attempts have been made to identify alternative effective anticancer drugs derived from its molecular structure. These efforts have involved the preparation and biological evaluation of many semi-synthetic derivatives of geldanamycin, often involving modifications at the C-17 position [39,40]. Despite these attempts, an anticancer agent based on the geldanamycin pharmacophore has yet to be approved for clinical use [41]. However, previous research has reported the production of 17-O-demethylgeldanamycin (**2**) by *Streptomyces* DEM20745. This is of particular interest due to its potential as a starting material for the development of new semi-synthetic geldanamycin derivatives for clinical evaluation (41). For example, 17-arylgeldanamycins, synthesized via a triflation/Suzuki coupling approach using synthetic 17-O-demethylgeldanamycin, have been shown to have potent inhibition of Hsp90 [42]. Nevertheless, the limited availability of 17-O-demethygeldanamycin (**2**) has restricted further synthetic work in this area [43]. Therefore, it is of interest that our results revealed the production of 17-O-demethyl-geldanamycin (**2**) as a natural product by *Streptomyces* RCU-064^T^, which provides future development of a production strain for this potentially valuable compound.

Reblastatin (**3**) and 17-Demethoxyreblastatin (**4**) are phenolic analogues of geldanamycin. Screening for novel compounds with the ability to inhibit phosphorylation of the retinoblastoma protein revealed reblastatin (**3**) [44], isolated as a minor constituent from the cultivation of *Streptomyces hygroscopicus*, which is also known for producing the potent Hsp90 disruptor geldanamycin (**1**) [31,45]. In the original study, reblastatin (**3**) demonstrated significant inhibition of the proliferation of the human histiocytic lymphoma U-937 cell line, with an IC_50_ value of 0.43 μg/mL [44]. Furthermore, it was found to exhibit a potent inhibitory activity in a cell-based oncostatin M signaling assay, with an IC_50_ value of 0.16 μM [46].

17-Demethoxyreblastatin (**4**) is a derivative of reblastatin (**3**), a natural product originally isolated from *Streptomyces* species. It is structurally related to reblastatin (**3**), differing in the presence of a demethoxy group at the C-17 position [45]. This modification alters the chemical properties and potentially affects the biological activities of the compound. Evaluations of its cytotoxic potential have revealed inhibitory effects on cancer cell growth and proliferation, similar to those observed with reblastatin (**3**). However, some research findings indicate that 17-demethoxyreblastatin (**4**) displayed unique biological characteristics when compared to reblastatin (**3**). The specific potency, selectivity, and mechanisms of action may vary between these two compounds due to the structural alteration [47].

Some *Streptomyces* strains have been reported to produce reblastatin (**3**) and 17-demethoxyreblastatin (**4**). The extraction of crude compounds from *Streptomyces hygroscopicus* JCM4427 using ethyl acetate, followed by purifying using octadecyl silica column chromatography and HPLC, revealed the presence of reblastin (**3**) and 17-demethoxyreblastatin (**4**) that exhibited Hsp90 ATPase inhibition activity with IC_50_ values of 0.32 μM and 1.82 μM, respectively, [48]. Despite 17-demethoxyreblastatin (**4**) being a derivative of reblastatin (**3**), there is limited literature available regarding its production from *Streptomyces* species. This scarcity of reports can be attributed to variations in the expression of the BGCs among different *Streptomyces* species [32].

Nocardamine (**5**), also called desferrioxamine, is a cyclic peptide siderophore found in several species of bacteria, including *Nocardia, Pseudomonas*, and *Streptomyces* species [49]. Siderophores are compounds synthesized by microorganisms to scavenge and bind iron from the surrounding environment. They act as an iron chelator, facilitating the uptake and utilization of this crucial nutrient by bacteria that require it for their growth and viability. Siderophores exhibit a strong affinity for iron and form stable complexes with the metal, effectively preventing its precipitation or interaction with other molecules present in the environment. Clinically, the nocardamine-type siderophore, Desferol, is used for the treatment of iron intoxication [50,51,52]. In addition, dehydroxynocardamine (**6**) is a derivative that is structurally related to nocardamine (**5**), with the key difference being the absence of hydroxyl groups in its chemical structure [53].

Nocardamine (**5**) was originally isolated as an antibacterial metabolite from a *Nocardia* strain [52], while nocardamine (**5**) and dehydroxynocardamine (**6**) were isolated from the culture broth of a marine-derived *Streptomyces* isolated from a sponge. Later, both compounds were also reported from soil *Streptomyces* sp. strain TS-2-2 and *Streptomyces* sp. BCC71188 [37,54]. This indicates that the ability to produce nocardamine (**5**) could be found in both terrestrial and marine *Streptomyces* species. In this study, nocardamine (**5**) and dehydroxynocardamine (**6**) were both inactive in the antimicrobial activity test at a concentration of 50 μg/mL, in accord with a previous report [49] that found no antimicrobial activity of both compounds at a final concentration of up to 200 μg/mL. However, in another study, nocardamine (**5**) showed a weak antimicrobial activity against *E. faecium* and *B. subtilis* but no activity against *V. alginolyticus* and *C. albicans* [55]. Moreover, in the same study, nocardamine (**5**) did not inhibit cell proliferation of tumor cell lines, including T-47D, SK-Mel-5, SK-Mel-28, and PRMI-7951, but did inhibit their colony formation [55]. Furthermore, the genetic engineering of *Streptomyces atratus* SCSIO ZH16, a deep-sea-derived *Streptomyces*, by in-frame deletion to activate putative orphan gene clusters, led to the production of new compounds, including nocardamine (**5**) [52].

In the analysis of BGCs, the detection of NRPS-like and T1PKS similar to the previous known BGCs of geldanamycins and the detection of desferrioxamine in the genome of strain RCU-064^T^ supported that strain RCU-064^T^ is the producer of geldanamycins (**1**) and nocardamines (**5**). This highlights the remarkable capacity of this genus to function as a potential source for drug production and a rich source of bioactive metabolites. The discovery and production of actinobacterial metabolites has been facilitated by advances in genome sequencing, bioinformatics, and genetic engineering techniques. These approaches have enabled the identification and manipulation of the BGCs responsible for compound synthesis, allowing researchers to optimize system production, enhance yields, and generate novel derivatives with improved properties. Moreover, the exploration of diverse environments, such as soil, marine sediments, and plant-associated microbiomes, has led to the discovery of novel Actinobacterial strains and expanded the diversity of known actinobacterial metabolites.

## 4. Materials and Methods

### 4.1. Microorganisms

Soil samples were collected from Nong Jum Rung peat swamp forest, Rayong Province, Thailand, in June 2011. *Streptomyces*, including the new isolate *S. rugosispiralis* sp. nov. (RCU064^T^), were isolated following the serial dilution method. Briefly, 1 g of soil sample was added into the basic lauryl sulfate buffer and 10-fold serially diluted to 10^−4^. Each dilution (0.1 mL) was spread on humic acid vitamin agar supplemented with nalidixic acid (25 mg/L) and cycloheximide (50 mg/L) and incubated at 30 °C for 14 days. After incubation, the colony of strain RCU-064^T^ was transferred to ISP2 agar and maintained as a working culture. The pure culture was preserved in lyophilized tube for long term preservation. The type strain used for taxonomic comparison was obtained from the Japan Collection of Microorganisms (JCM) and maintained under the same condition used for strain RCU-064^T^.

### 4.2. Phenotypic Study

Morphological observation was performed under light microscopy on the culture grown on ISP2 medium at 30 °C for 14 days. The spore morphology and spore surface were observed using scanning electron microscopy (JSM-5401LV, JEOL, Tokyo, Japan). Cultural characteristics on agar media were determined according to the standard method [56]. The color of aerial mycelia, substrate mycelia, and soluble pigment was determined using the NBS/IBCC colour system [57]. Starch hydrolysis, gelatin liquefaction, skim milk peptonization, and nitrate reduction were determined using the reported methods [58,59]. Carbon utilization was determined following the previously reported method [56]. Enzyme activities were determined using API ZYM (bioMérieux), at 37 °C for 5 h. Growth temperature, pH, and NaCl tolerance were determined on ISP2 medium.

Isomers of diaminopimelic acid, whole-cell sugars, and the presence of mycolic acid were determined using standard thin layer chromatography (TLC) as reported [60,61]. Menaquinones were extracted following [62] and were analyzed using HPLC. *N*-Acyl type of muramic acid was determined as reported [63]. Cellular fatty acids were prepared and analyzed using gas chromatography–mass spectrometry following the MIDI Sherlock Microbial Identification System [64]. Phospholipids were extracted and determined using two-dimensional TLC [65].

### 4.3. 16S rRNA Gene and Phylogeny

Genomic DNA were obtained from freeze-dried cells following [66] and used for the PCR amplification of 16S rRNA gene as previously reported [67]. The nucleotide sequencing of the PCR products (Macrogen, Seoul, Republic of Korea) was determined using universal primers [68]. BLAST analysis was performed using EzTaxon-e server [69]. The sequence was aligned against the top 48 highest 16S rRNA gene similarity type strains obtained from the GenBank/EMBL/DDBJ database using BioEdit software [70]. The ML phylogenetic trees were constructed using MEGA version 7.0 [71] and the topologies of the resultant trees were evaluated using bootstrap resampling with 1000 replications.

### 4.4. Genome and Bioinformatics

The genomic DNA of strains RCU-064^T^ was extracted from the cells grown in ISP2 broth for 5 days using a DNA extraction kit (PureLink, Invitrogen, Thermo Fisher Scientific, Waltham, MA, USA). The QIAGEN FX kit was used for preparing the DNA library. Quality and quantity of the indexed libraries were analyzed using Agilent 2100 Bioanlyzer and Denovix fluorometer and pooled in an equimolar quantity. Cluster generation and paired-end 2 × 150 nucleotide read sequencing were performed on Illumina HiSeq X ten sequencer. FASTQC (Babraham Bioinformatics) was used for checking the quality of the raw read. Trim Galore was used to eliminate the low quality read and adaptors. PATRIC web service was used to assemble and annotate the genome [72]. The assembled genome of strain RCU-064^T^ was deposited at DDBJ/ENA/GenBank under accession number JANIAA000000000. Prediction of the BCGs in the genome was analyzed using anti-SMASH version 5.0 [73]. Average nucleotide identity (ANI) values were determined using JSpecies web service [74]. Digital DNA-DNA hybridization (dDDH) value was calculated using the Genome-to-Genome Distance Calculator (GGDC 2.1) [75]. The phylogenomic tree was constructed using AutoMLST [76].

### 4.5. Fermentation and Isolation of Secondary Metabolites

The culture plates of strain RCU-064^T^ were used for the stock culture, which were grown on ISP2 agar medium at 30 °C for 4 days. The stock culture was inoculated into 250-mL Erlenmeyer flasks containing 150 mL of ISP2 broth (seed medium). The seed culture of strain RCU-064^T^ was cultivated on a rotary shaker (200 rpm) at 30 °C for 4 days. Then, the seed culture (20 flasks) was transferred into 80 × 1-L Erlenmeyer flasks, each containing 250 mL of ISP 2 broth and cultivated at 30 °C on a rotary shaker (200 rpm) for 14 days. After that, the culture was extracted three times with an equal volume of ethyl acetate, then the pooled extracts were dried over Na_2_SO_4_ and then evaporated to dryness to yield the crude extract (5.8 g). The crude extract was fractionated through a Sephadex LH-20 column to give three fractions (F1–F3).

Fraction F1 (2.5 g) was enriched using preparative HPLC, eluting with a linear gradient system of 30–95% acetonitrile (CAN) in water (over 40 min at a flow rate of 15 mL/min, to furnish compounds (**1**) (72.6 mg) and (**2**) (44.4 mg).

Fraction F2 (0.8 g) was re-separated by Sephadex LH-20 column chromatography to give two sub-fractions (F2F1 and F2F2). Sub-fractions F2F1 (0.1 g) and F2F2 (0.7 g) were each fractionated by preparative HPLC, eluting with a linear gradient system of 30–95% ACN in H_2_O over 40 min at a flow rate of 15 mL/min, to give compounds (**5**) (8.2 mg) and (**2**) (3.1 mg) from F2F1 and compounds (**5**) (68.2 mg) and (**6**) (34.7 mg) from F2F2.

The final major fraction, F3 (2.2 g) was fractionated by Sephadex LH-20 column chromatography to give three sub-fractions (F3F1, F3F2, and F3F3). Each of these sub-fractions were fractioned as per subfractions F2 above to give compound (**4**) (20.1 mg) from F3F1 (0.1 g), compounds (**3**) (47.8 mg) and (**4**) (54.2 mg) from F3F2 (0.2 g), and compounds (**1**) (120.1 mg) and (**2**) (90.2 mg) from F3F3 (1.1 mg). The isolation and enrichment procedure of these compounds is summarized in Figure 4.

### 4.6. Characterization and Identification of Secondary Metabolites

The NMR spectra were recorded on Bruker Avance 500 MHz and Bruker Avance III 400 MHz NMR spectrometers using acetone-*d_6_* as an internal standard, while HRESIMS data were obtained from a Bruker MicOTOF spectrometer. Column chromatography was performed on a Sephadex LH-20 column using 100% methanol as the eluent. HPLC was performed on a Dionex-Ultimate 3000 series equipped with a binary pump, an autosampler, and diode array detector. Semi-preparative HPLC was performed on a Sunfire C18 column from Waters (5 µm, diam. 19 mm × 150 mm). Preparative HPLC was performed on a Sunfire C18 column from Waters (10 µm, diam. 19 mm × 250 mm).

### 4.7. Biological Activity

Antibacterial activity was tested using the resazurin microplate assay (REMA) [77]. The MIC represents the lowest concentration that inhibited growth of the tested bacteria. Vancomycin was used as a positive control. The green fluorescent protein microplate assay (GFPMA) was used for evaluation of cytotoxicity against Vero cells (African green monkey kidney fibroblasts, ATCC CCL-81) and antituberculosis activity against *Mycobacterium tuberculosis* strain H37Ra [78]. Ellipticine and amphotericin B were used as the positive controls for cytotoxicity against Vero cells and anti-phytopathogenic activity, respectively. For antituberculosis, isoniazid, ofloxacin, rifampicin, streptomycin, and ethambutol were used as the positive controls. Antimalarial assay against *Plasmodium falciparum* (K-1, multi-drug resistant strain) was performed using the microculture radioisotope technique [79]. The IC_50_ value represents the concentration that caused a 50% reduction in parasite growth. Dihydroartemisinin and mefloquine were used as the positive controls. Cytotoxicity against KB (oral human epidermoid carcinoma, ATCC CCL-17), MCF-7 (human breast cancer, ATCC HTC-22), and NCI-H187 (human small-cell lung cancer, ATCC CRL-5804) cell lines were evaluated using the resazurin microplate assay (REMA) as previously described [80]. Ellipticine and doxorubicin were used as the positive controls for anti-KB, with tamoxifen and doxorubicin as the positive controls for anti-MCF-7 and doxorubicin was used as the positive control for anti-NCI-H187. The NA inhibition assay was determined using the MUNANA-based enzyme inhibition assay [81].

## Figures and Tables

**Table 1:** ANIb and ANIm values (%) and the digital DNA-DNA hybridization (dDDH) values between the draft genomes of strain RCU-064^T^ and its related *Streptomyces* type strains.

Strains	Genome Size (nt)	Accession Number	% GC Content	% dDDH	ANIb	ANIm
RCU-064^T^	11017810	GCA_024505145	71.3	-	-	-
*S. malaysiensis* DSM4137^T^	10744231	GCA_002591335	71.1	43.8	90.2	92.1
*S. samsunensis* DSM 42010^T^	11053136	GCF_024519315	70.9	43.5	89.3	92.1
*S. melagnosporofaciens* DSM 40318^T^	10769732	GCA_900105695	71.0	43.2	89.5	91.9

**Table 2:** The distribution of secondary metabolites biosynthetic gene clusters in the genome of strain RCU-064^T^. Only biosynthetic gene clusters with similarity of greater than 50% are shown.

Cluster	Type	Most Similar Known Cluster (Class)	Similarity (%)
1	aryl polyene, ladderane, NRPS, aminocoumarin	Coprisamide C/coprisamide D	95%
2	CDPS	bicyclomycin	100%
3	terpene	pristinol	100
4	NRPS-like, T1PKS	Geldanamycin	69
5	Terpene	2-methylisoborneol	100
6	T1PKS	efomycin K/efomycin L	100
7	T1PKS	ectoine	100
8	NRPS-like	echoside A/echoside B/echosieC/echosideD/echoside E	100
9	T2PKS	spore pigment	83
10	NI-siderophore	Legonoxamine A/desferrioxamine B/legonoxamine B	66
11	Ladderane, aryl polyene	o-dialkylbenzene 1/o-dialkylbenzene 2	61
12	T3PKS, NRPS	feglymycin	84
13	T1PKS	nigericin	63
14	terpene	Hopene	53
15	T1PKS	spirangien O	53

**Table 3:** Cellular fatty acid composition of strain RCU-064^T^.

Fatty Acid	RCU-064^T^
Saturated fatty acids	
C_14:0_	2.7
C_16:0_	14.4
C_17:0_ cyclo	5.1
Branched fatty acids	
iso-C_14:0_	8.5
iso-C_15:0_	8.6
anteiso-C_15:0_	13.0
iso-C_16:1_H	3.1
iso-C_16:0_	21.4
iso-C_17:0_	2.3
anteiso-C_17:0_	4.1
Unsaturated fatty acids	
anteiso-C_17:1_ω9c	2.2
Sum in feature 3 ^a^	3.2
Sum in feature 9 ^b^	2.5

^a^ Summed feature 3 comprised C_16:1_ω7c and/or _C16:1_ω6c. ^b^ Summed feature 8 comprised C_16:0_10-methyl or iso-C_17:1_ω9c.

**Table 4:** Cultural characteristics of strain RCU-064^T^.

Culture Media	Growth	Aerial Masses Color	Substrate Mycelia Color	Soluble Pigment
ISP2	Very good	White, light greenish-grey	Dark greenish-yellow	-
ISP3	Very good	White	Dark greenish-yellow	-
ISP4	Good	White, grey	Greyish-yellow	-
ISP5	Good	White	Pale greenish-yellow	-
ISP6	Good	Absent	Greyish-yellow	-
ISP7	Good	White	Greyish-yellow	-
Nutrient agar	Good	White, grey	Greyish-yellow	-

**Table 5:** Differential characteristics between strain CU-064^T^ and its closely related *Streptomyces* type strains. * and ** were obtained from [17] and [18], respectively. +, positive; − negative; nd, not determined.

Phenotypic Properties	Strain			
	CU-064^T^	*S. malaysiensis* JCM 10672^T^	*S. samsunensis* M1463^T^ *	*S. melanosporofaciens* ISP 5318^T^ **
Liquefy gelatin	−	+	−	nd
Skim milk peptonization	−	+	nd	nd
Nitrate reduction	−	+	+	nd
NaCl tolerance (% [*w*/*v*])	6%	5%	nd	nd
Carbon utilization of:				
Arabinose	+	+	+	nd
Cellobiose	++	++	+	nd
*myo*-inositol	+	+	−	+
Salicin	±	+	−	nd
Raffinose	++	+	−	+
Fructose	±	++	+	+
Sucrose	−	−	−	−

**Table 6:** Biological activity of isolated compound from strain RCU-064^T^.

Compound	Anti-*B. cereus* MIC (µg/mL)	Anti-*M. tuberculosis* MIC (µg/mL)	Anti-*P. falciparum* IC_50_ (µg/mL)	Cytotoxicity IC_50_ (µg/mL)			
				Vero	KB	NCI-H187	MCF-7
(**1**)	inactive	inactive	inactive	0.094	16.72	0.045	3.510
(**2**)	inactive	inactive	inactive	6.430	inactive	4.250	inactive
(**3**)	inactive	inactive	inactive	7.750	21.30	0.313	1.270
(**4**)	inactive	inactive	inactive	13.50	inactive	1.330	inactive
(**5**)	inactive	inactive	inactive	16.57	inactive	inactive	inactive
(**6**)	inactive	inactive	inactive	inactive	inactive	13.960	inactive
Vancomycin *	2.00	-	-	-	-	-	-
Rifampicin *	-	0.013	-	-	-	-	-
Ofloxacin *	-	0.391	-	-	-	-	-
Isoniazid *	-	0.047	-	-	-	-	-
Ethambutol *	-	0.938	-	-	-	-	-
Dihydroartemisinine *	-	-	7.02 × 10^−4^	-	-	-	-
Mefloquine *	-	-	0.024	-	-	-	-
Ellipticine *	-	-	-	1.580	1.810	1.65	-
Doxorubicin *	-	-	-	-	0.655	0.088	8.07
Tamoxifen *	-	-	-	-	-	-	6.96

* = positive control; -, not determined. Inactive, at the final concentration of 50 μg/mL.

## Data Availability

The GenBank accession number of the 16S rRNA gene sequences and draft genome of strain RCU-064^T^ are OQ001562 and JANIAA000000000, respectively.

## Supplementary Materials

The following supporting information can be downloaded at: https://www.mdpi.com/article/10.3390/antibiotics12091467/s1, Spectroscopic data of compounds (**1**)–(**6**), including ^1^H- and ^13^C-NMR spectra, and mass spectra (see Figures S1–S18). Figure S1. ^1^H NMR spectrum (CDCl_3_) of compound (**1**); Figure S2. ^13^C NMR spectrum (CDCl_3_) of compound (**1**); Figure S3. ^1^H NMR spectrum (CDCl_3_) of compound (**2**); Figure S4. ^13^C NMR spectrum (CDCl_3_) of compound (**2**); Figure S5. ^1^H NMR spectrum (DMSO-*d*_6_) of compound (**3**); Figure S6. ^13^C NMR spectrum (DMSO-*d*_6_) of compound (**3**); Figure S7. ^1^H NMR spectrum (DMSO-*d*_6_) of compound (**4**); Figure S8. ^13^C NMR spectrum (DMSO-*d*_6_) of compound (**4**); Figure S9. ^1^H NMR spectrum (DMSO-*d*_6_) of compound (**5**); Figure S10. ^13^C NMR spectrum (DMSO-*d*_6_) of compound (**5**); Figure S11. ^1^H NMR spectrum (CDCl_3_) of compound (**6**); Figure S12. ^13^C NMR spectrum (CDCl_3_) of compound (**6**); Figure S13. MS spectrum of compound (**1**); Figure S14. MS spectrum of compound (**2**); Figure S15. MS spectrum of compound (**3**); Figure S16. MS spectrum of compound (**4**); Figure S17. MS spectrum of compound (**5**); Figure S18. MS spectrum of compound (**6**).

## Sample Availability

The type strain RCU-064^T^ is deposited at the culture collections of the Thailand Bioresource Research Center (TBRC) and Biological Resource Center, NITE (NBRC), Japan. Samples of compounds **1**–**6** are available from KS and PP.
