# Supplementary material for: Streptomyces rugosispiralis sp. nov., a Novel Actinobacterium Isolated from Peat Swamp Forest Soil That Produces Ansamycin Derivatives and Nocardamines
Source: Antibiotics (Basel). 2023 Sep 20;12(9):1467. doi: 10.3390/antibiotics12091467 (PMC10525797; doi:10.3390/antibiotics12091467)

Supplementary Materials

Figure S1. ^{1}H NMR spectrum ($CDCl_3$) of compound **(1)**

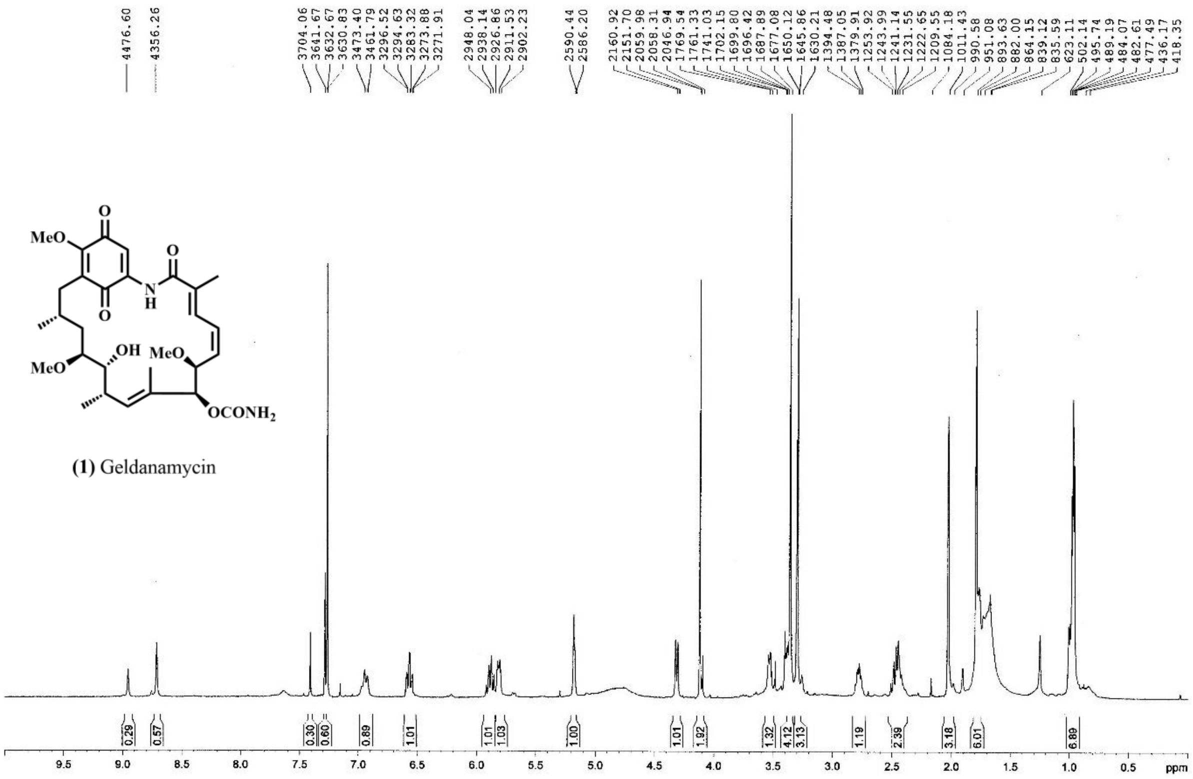

Figure S2. ^{13}C NMR spectrum ($CDCl_3$) of compound **(1)**

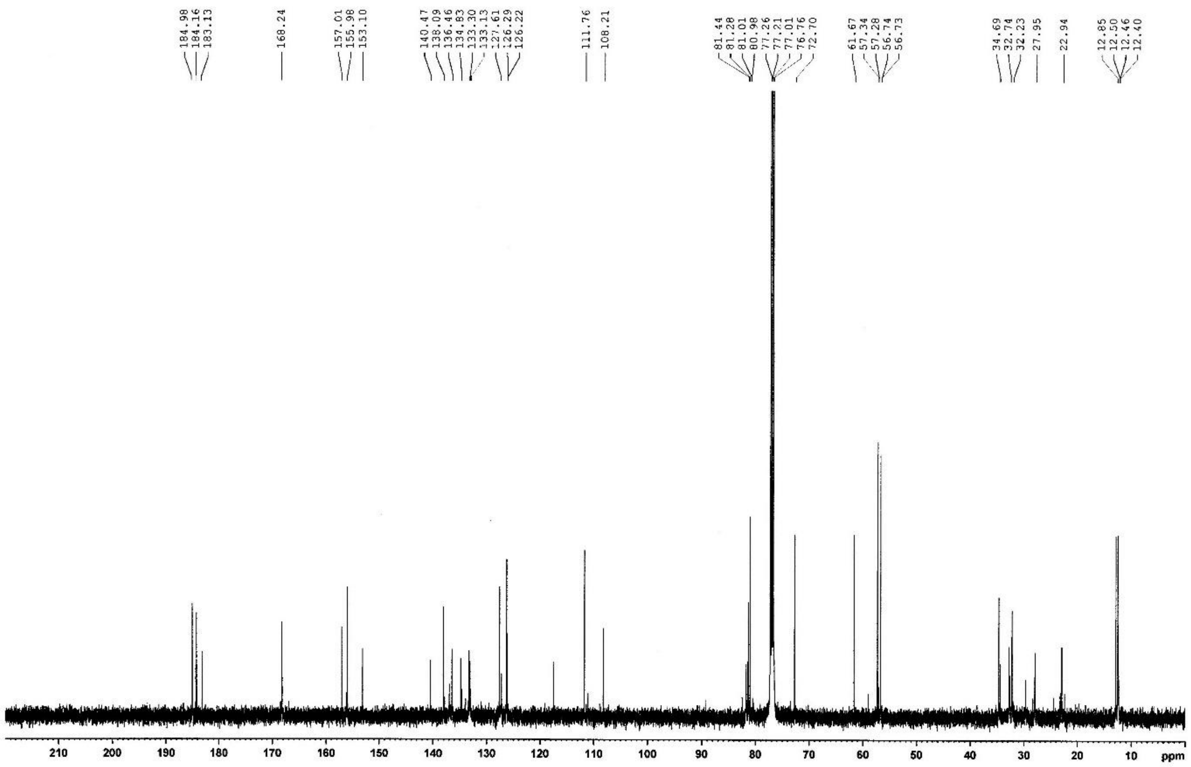

Figure S3. ^{1}H NMR spectrum ($CDCl_3$) of compound **(2)**

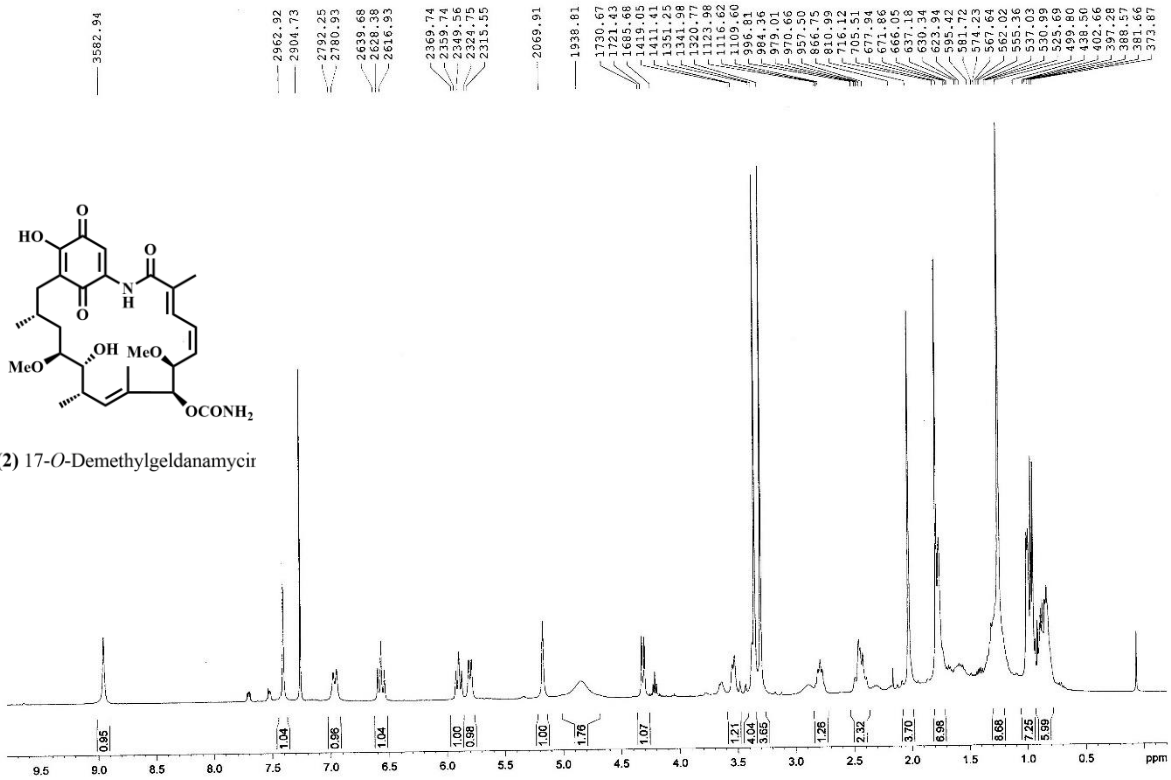

(2) 17-*O*-Demethylgeldanamycin

Figure S4. ^{13}C NMR spectrum ($CDCl_3$) of compound **(2)**

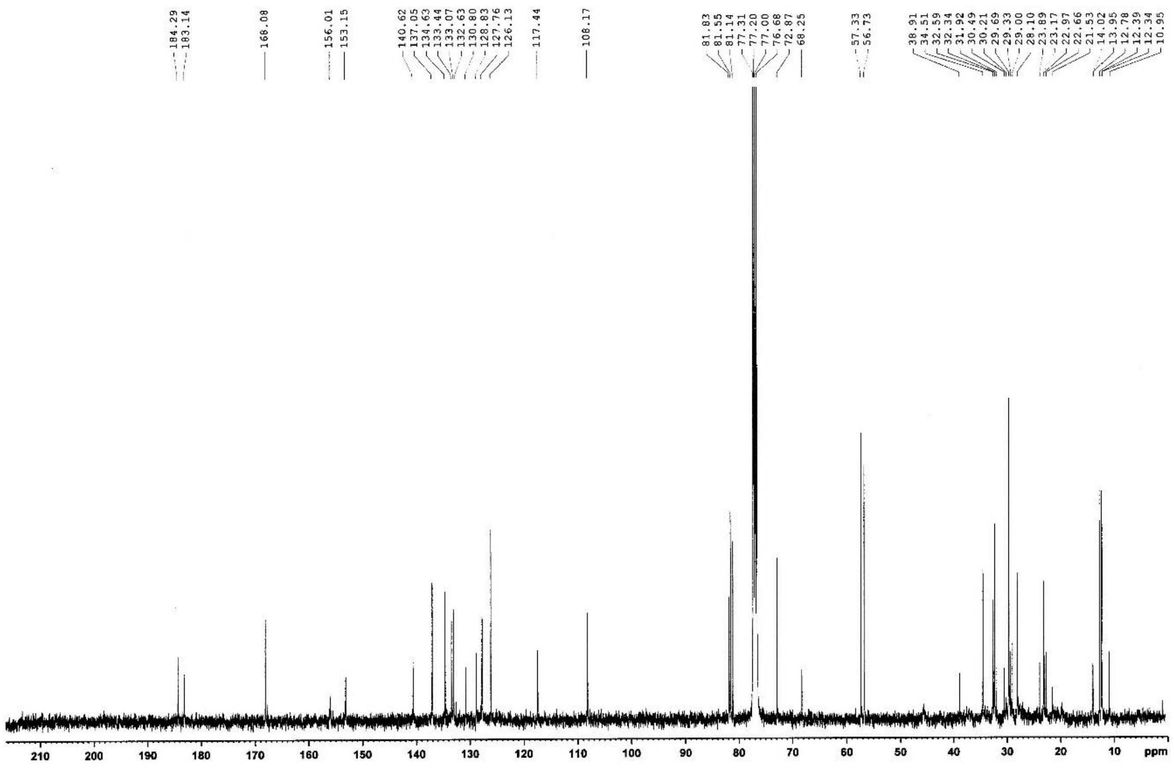

Figure S5. ^{1}H NMR spectrum (DMSO-d_6) of compound **(3)**

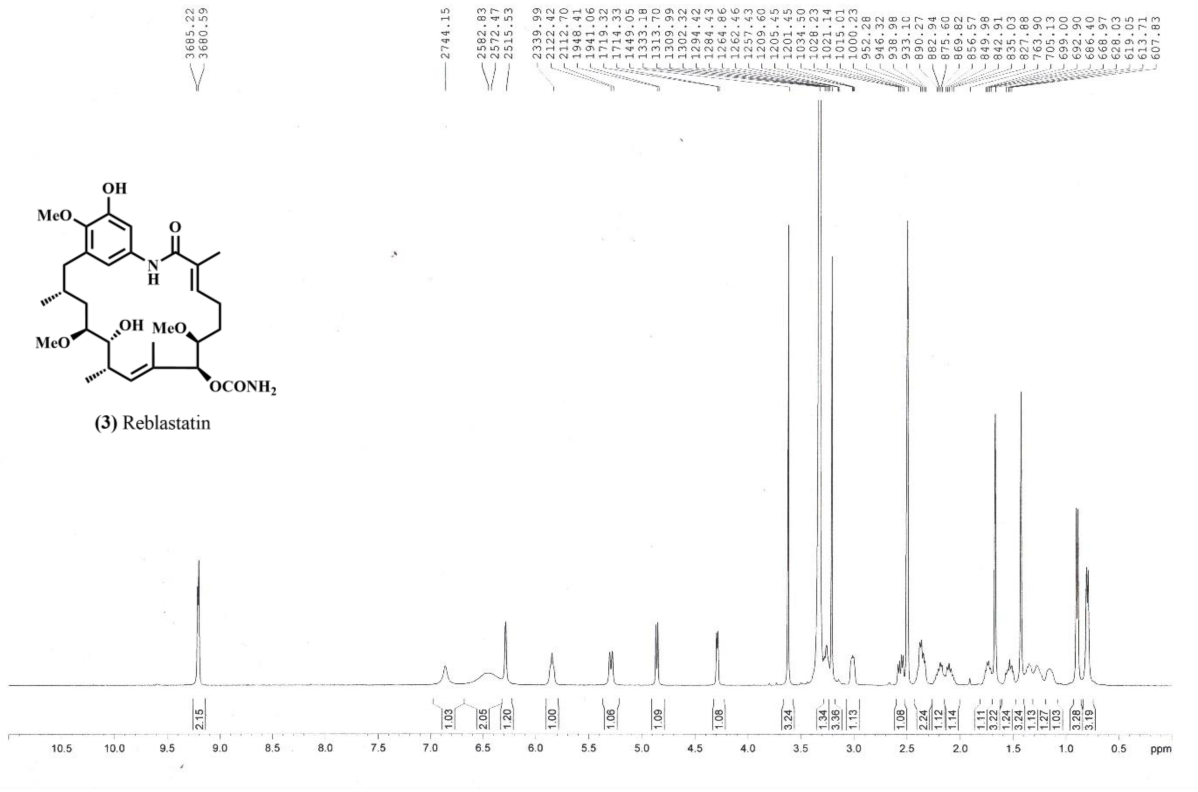

Figure S6. ^{13}C NMR spectrum (DMSO-d_6) of compound **(3)**

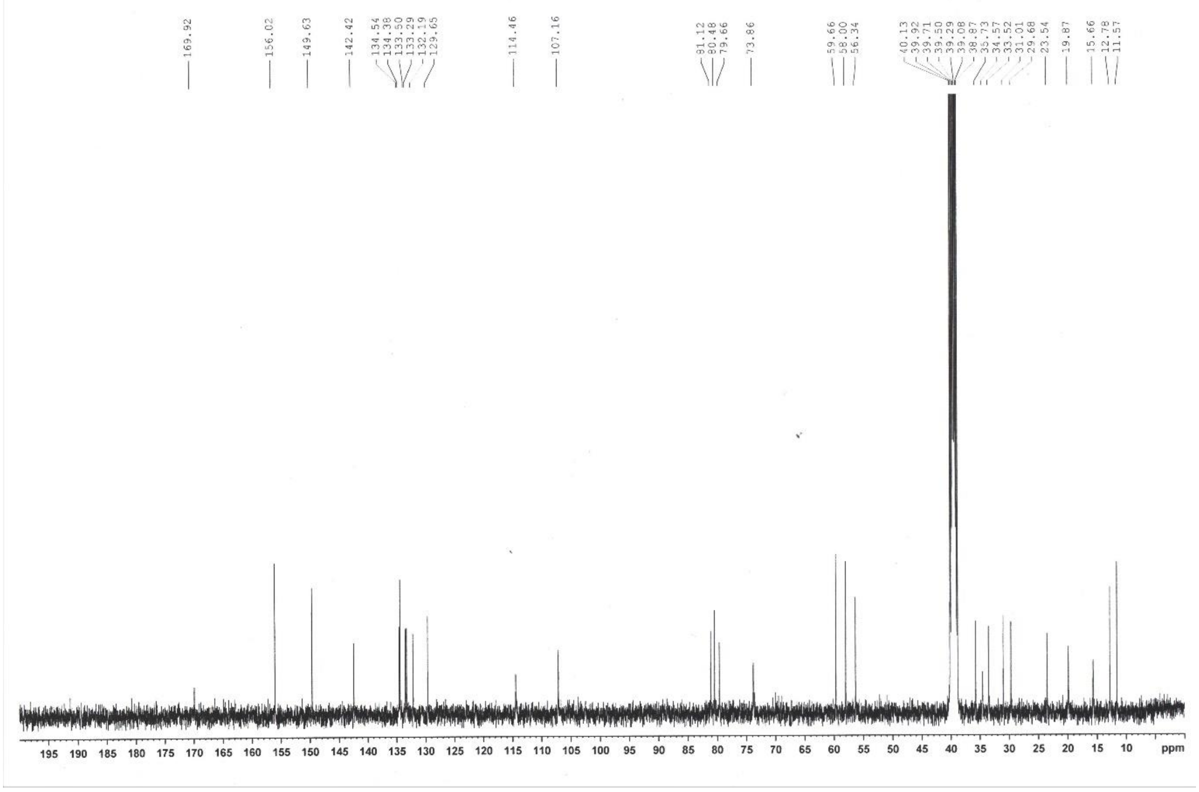

Figure S7. ^{1}H NMR spectrum (DMSO-d_6) of compound **(4)**

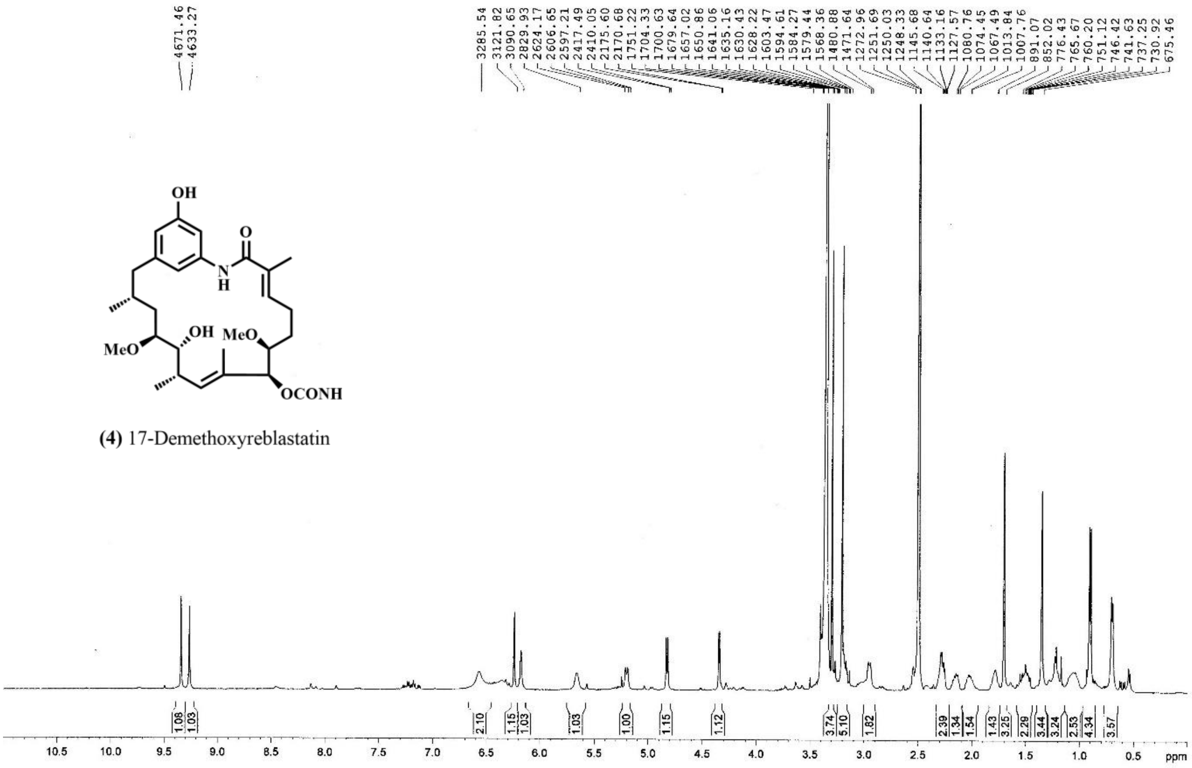

Figure S8. ^{13}C NMR spectrum (DMSO-d_6) of compound **(4)**

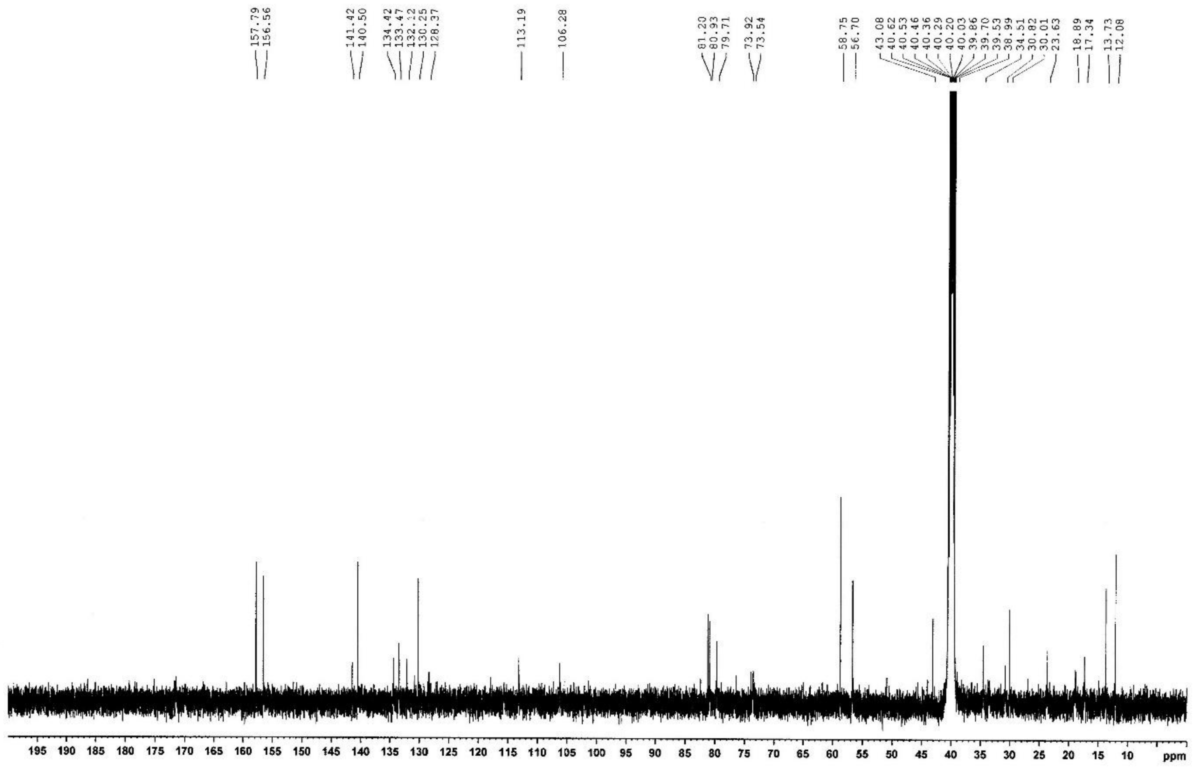

Figure S9. ^{1}H NMR spectrum (DMSO-d_6) of compound **(5)**

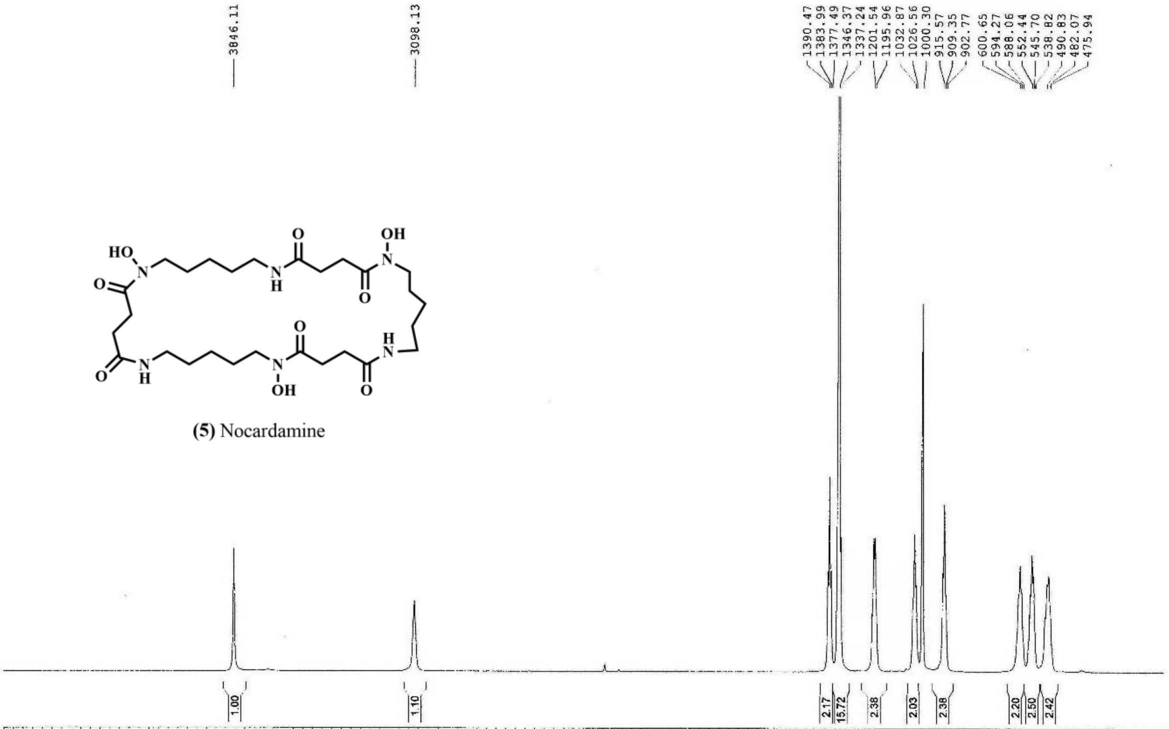

Figure S10. ^{13}C NMR spectrum (DMSO-d_6) of compound **(5)**

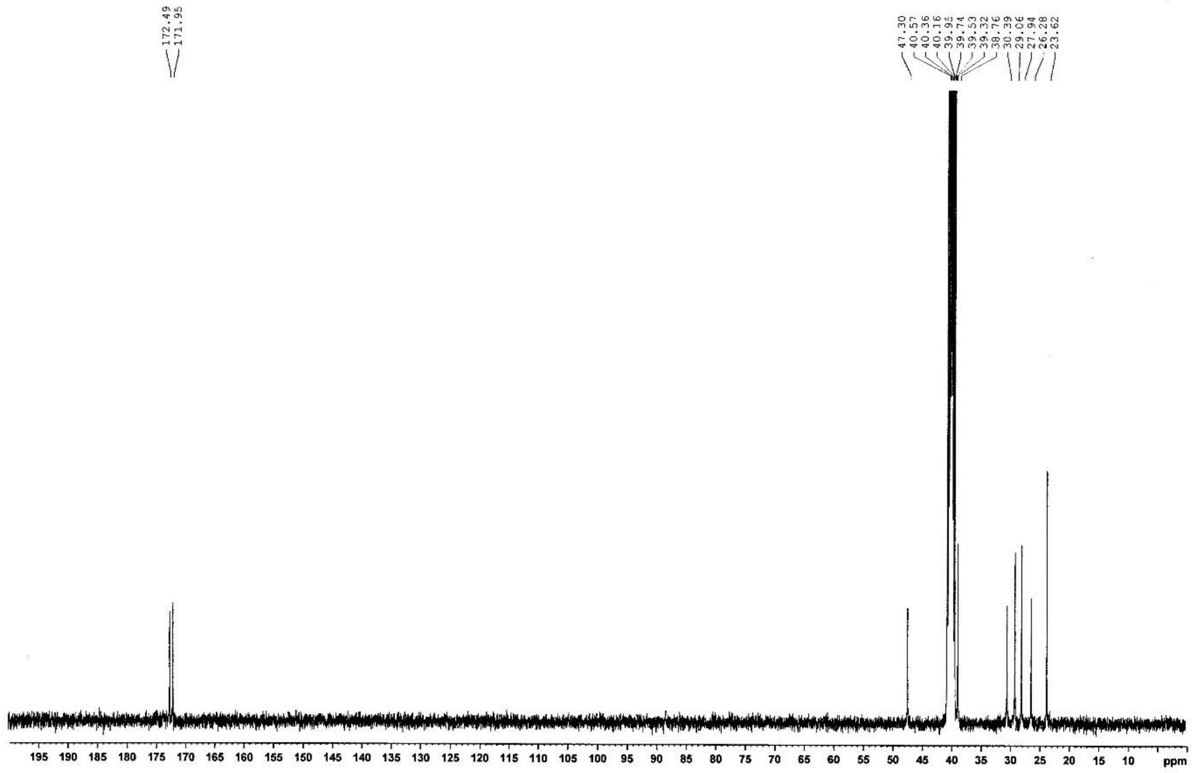

Figure S11. ^{1}H NMR spectrum ($CDCl_3$) of compound **(6)**

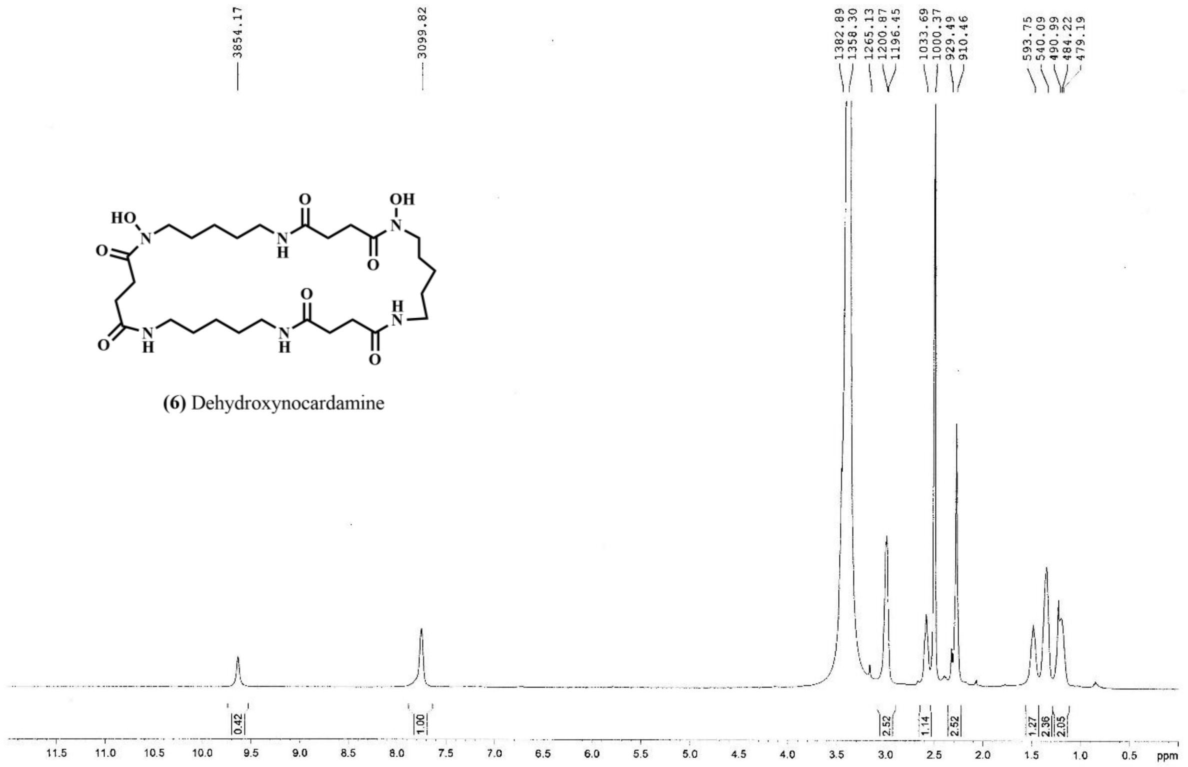

Figure S12. ^{13}C NMR spectrum ($CDCl_3$) of compound **(6)**

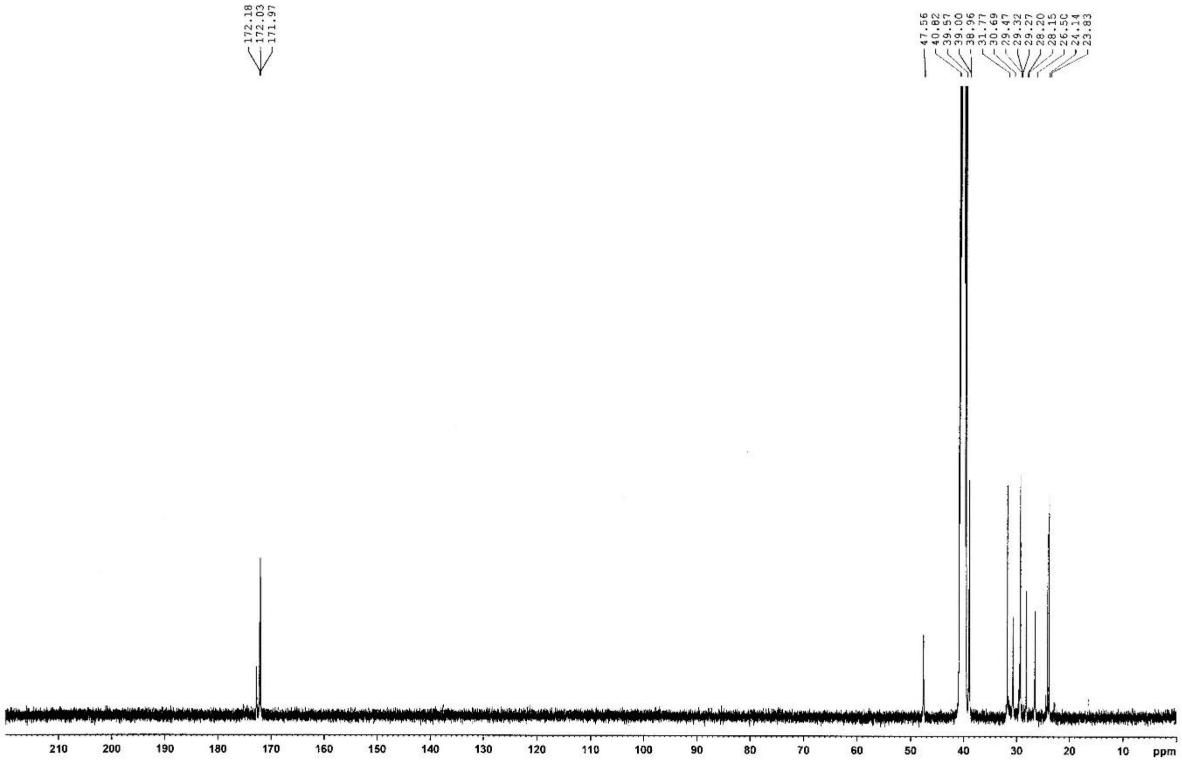

Figure S13. MS spectrum of compound **(1)**

Acquisition Parameter

Source Type	ESI	Ion Polarity	Negative	Set Nebulizer	0.4 Bar
Focus	Not active			Set Dry Heater	200 ?C
Scan Begin	50 m/z	Set Capillary	4500 V	Set Dry Gas	5.0 l/min
Scan End	1500 m/z	Set End Plate Offset	-500 V	Set Divert Valve	Source

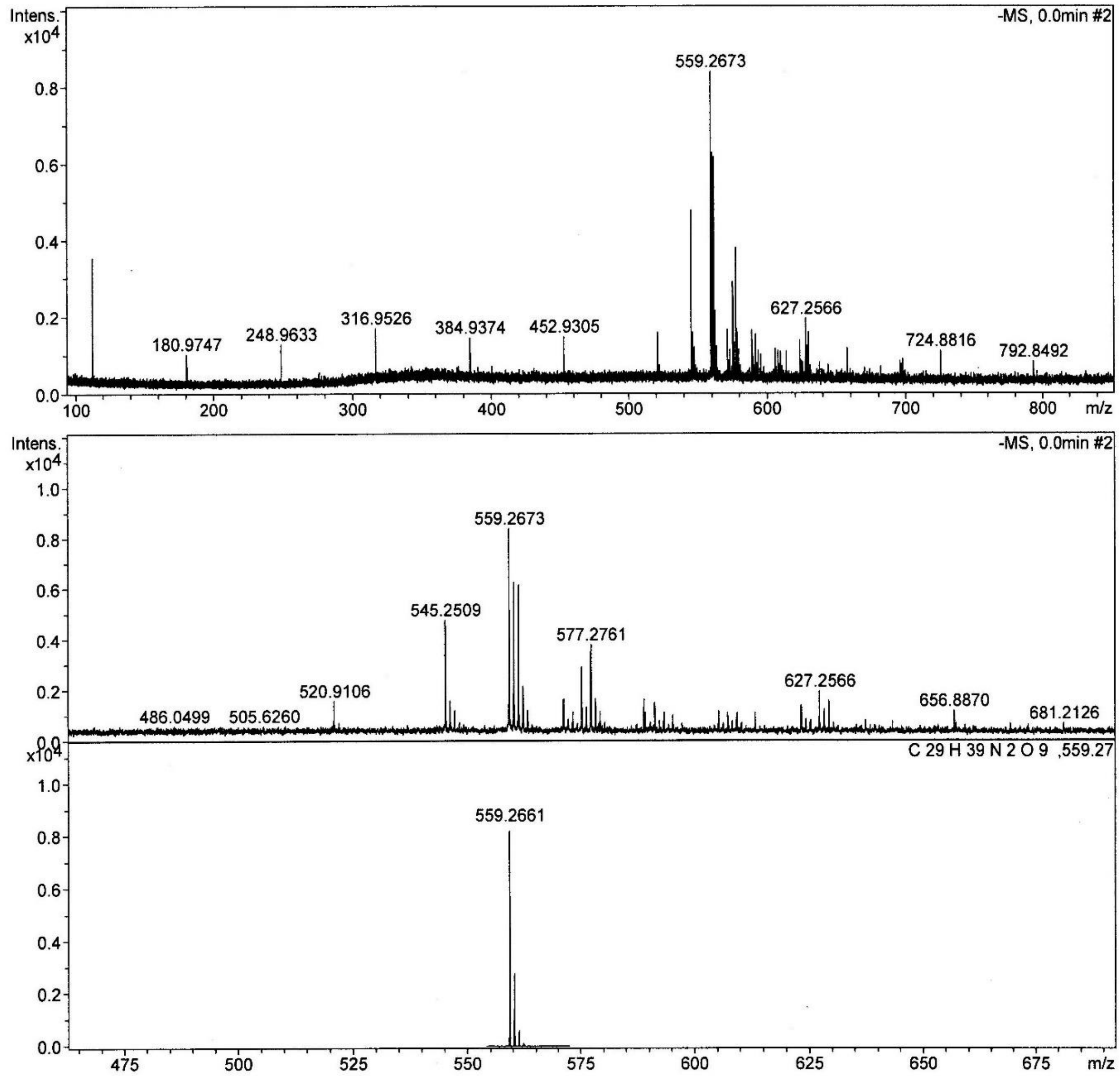

Figure S14. MS spectrum of compound **(2)**

Acquisition Parameter

Source Type	ESI	Ion Polarity	Negative	Set Nebulizer	0.4 Bar
Focus	Not active			Set Dry Heater	200 ?C
Scan Begin	50 m/z	Set Capillary	4500 V	Set Dry Gas	5.0 l/min
Scan End	1500 m/z	Set End Plate Offset	-500 V	Set Divert Valve	Source

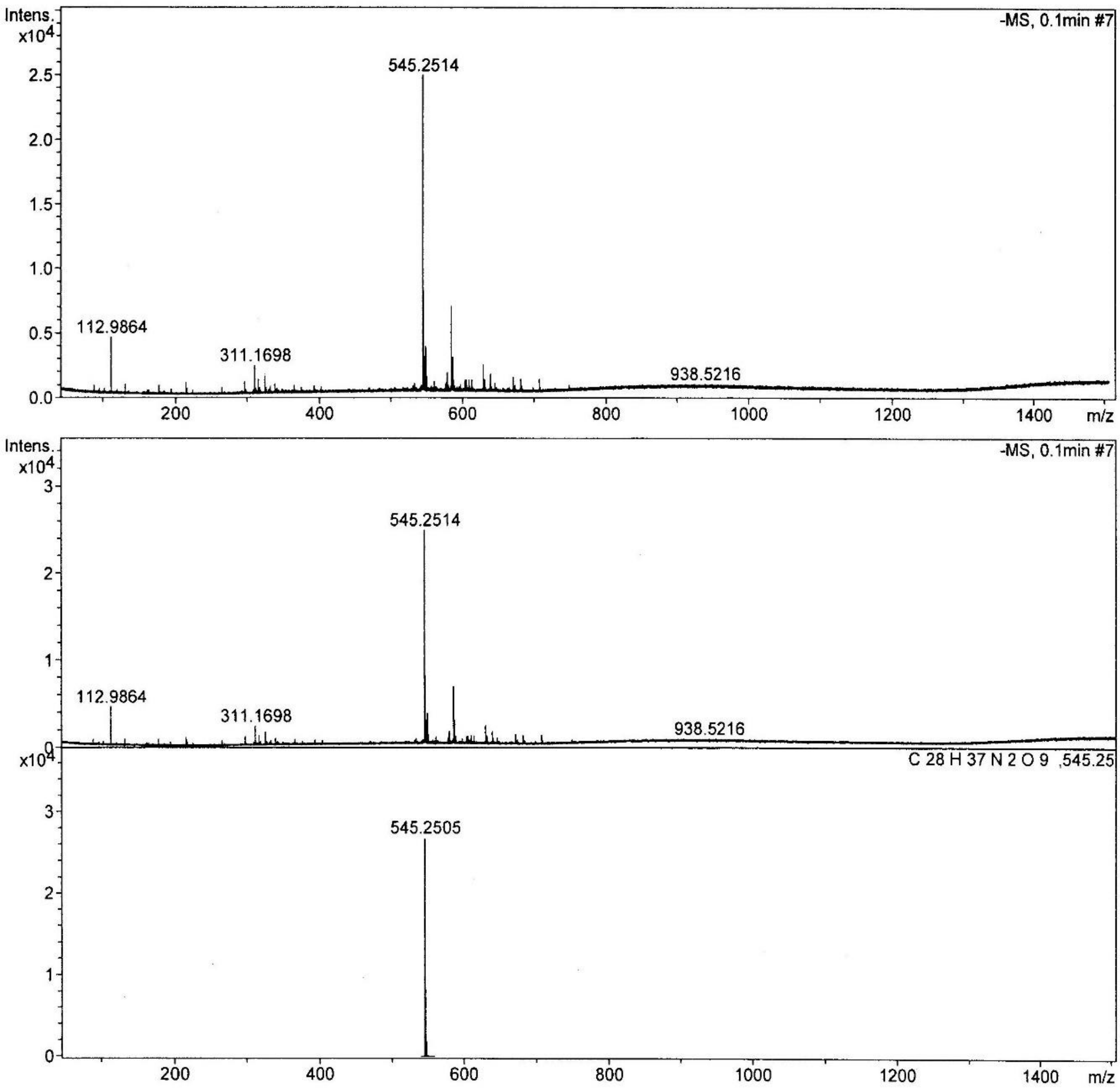

Figure S15. MS spectrum of compound **(3)**

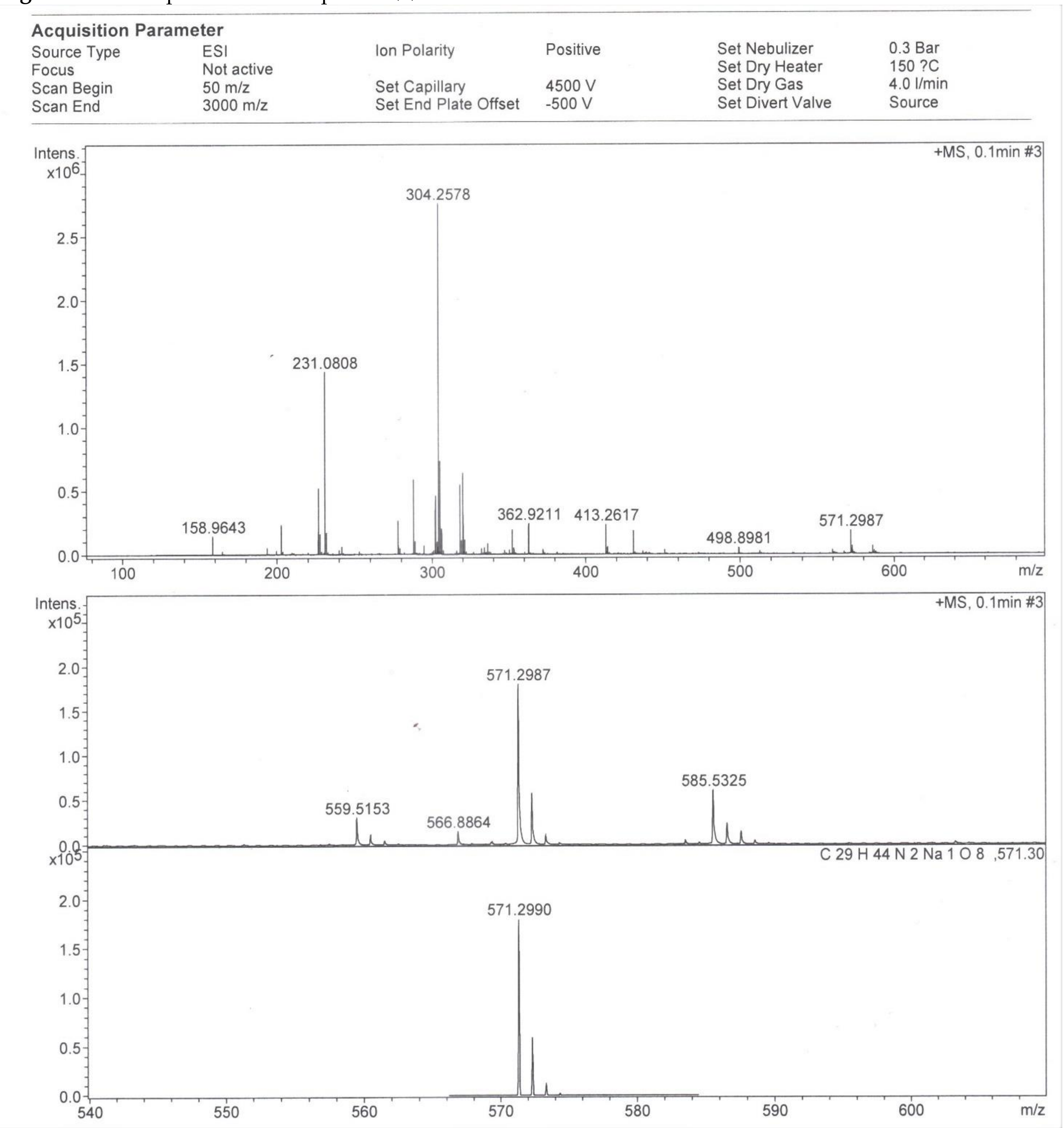

Figure S16. MS spectrum of compound **(4)**

Acquisition Parameter

Source Type	ESI	Ion Polarity	Positive	Set Nebulizer	1.0 Bar
Focus	Not active			Set Dry Heater	150 ?C
Scan Begin	100 m/z	Set Capillary	5000 V	Set Dry Gas	4.0 l/min
Scan End	1500 m/z	Set End Plate Offset	-500 V	Set Divert Valve	Source

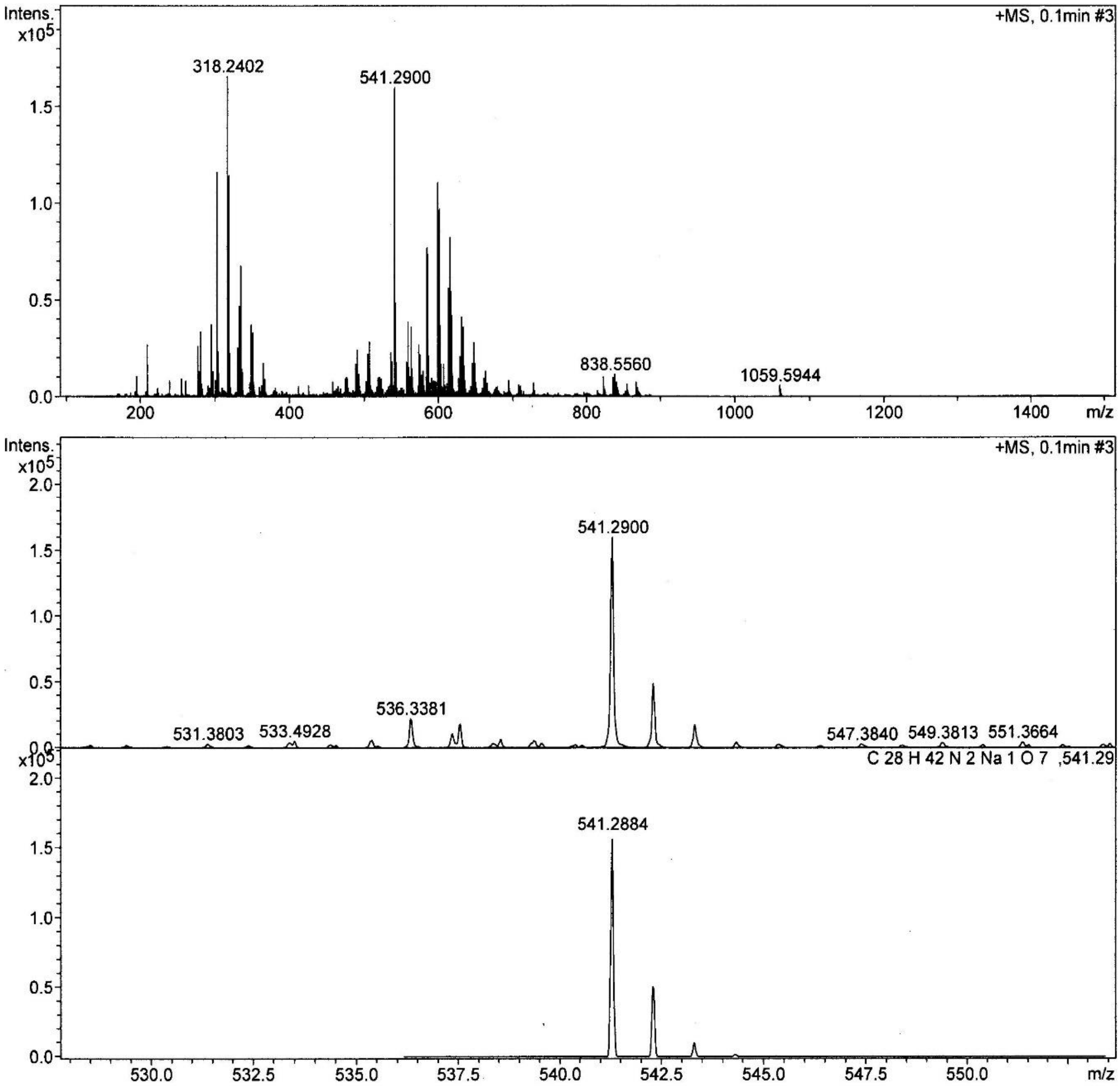

Figure S17. MS spectrum of compound **(5)**

Acquisition Parameter

Source Type	ESI	Ion Polarity	Positive	Set Nebulizer	0.3 Bar
Focus	Not active			Set Dry Heater	150 ?C
Scan Begin	50 m/z	Set Capillary	4500 V	Set Dry Gas	4.0 l/min
Scan End	3000 m/z	Set End Plate Offset	-500 V	Set Divert Valve	Source

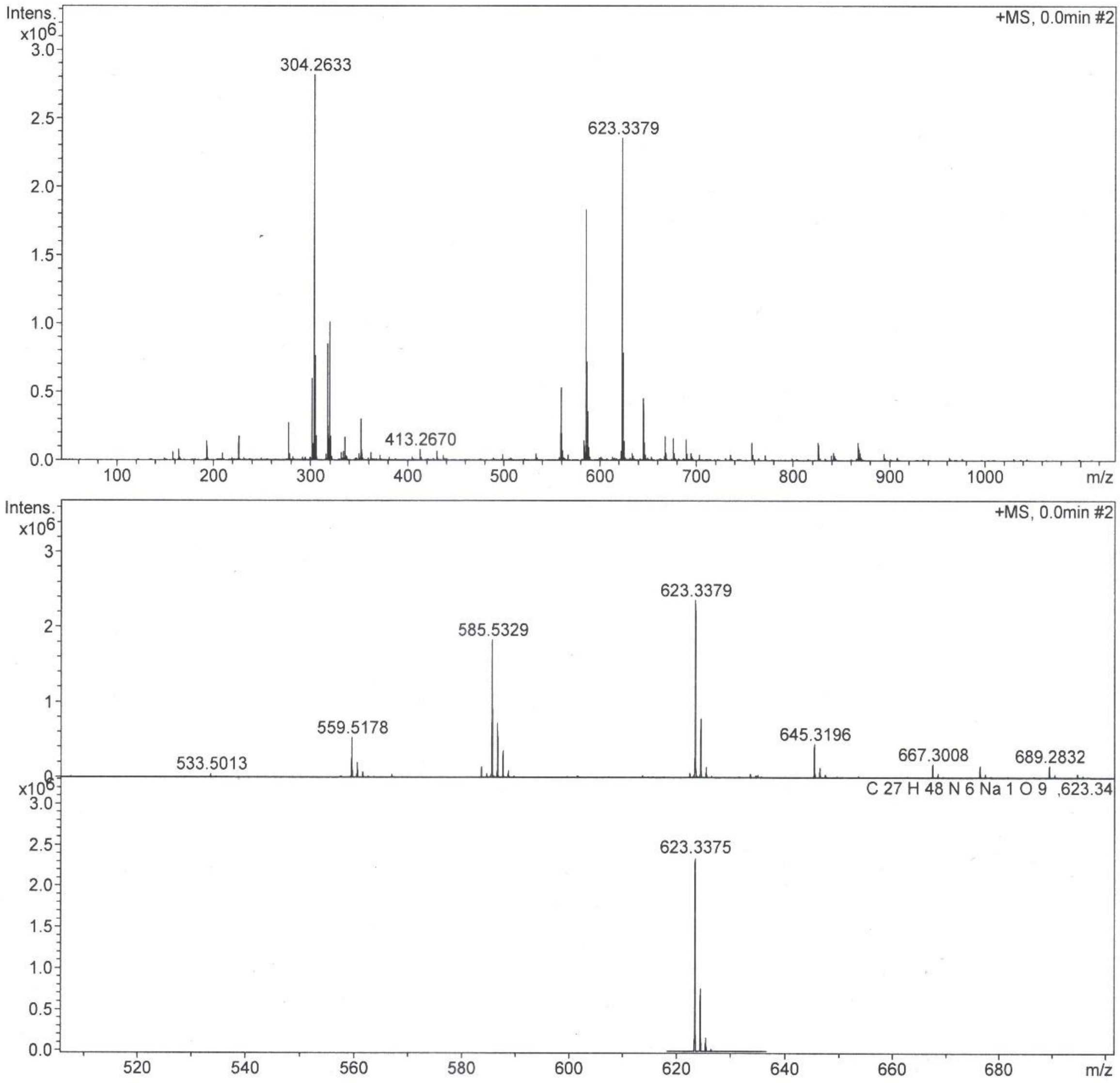

Figure S18. MS spectrum of compound **(6)**

Acquisition Parameter

Source Type	ESI	Ion Polarity	Positive	Set Nebulizer	1.0 Bar
Focus	Not active			Set Dry Heater	150 ?C
Scan Begin	100 m/z	Set Capillary	5000 V	Set Dry Gas	4.0 l/min
Scan End	1500 m/z	Set End Plate Offset	-500 V	Set Divert Valve	Source

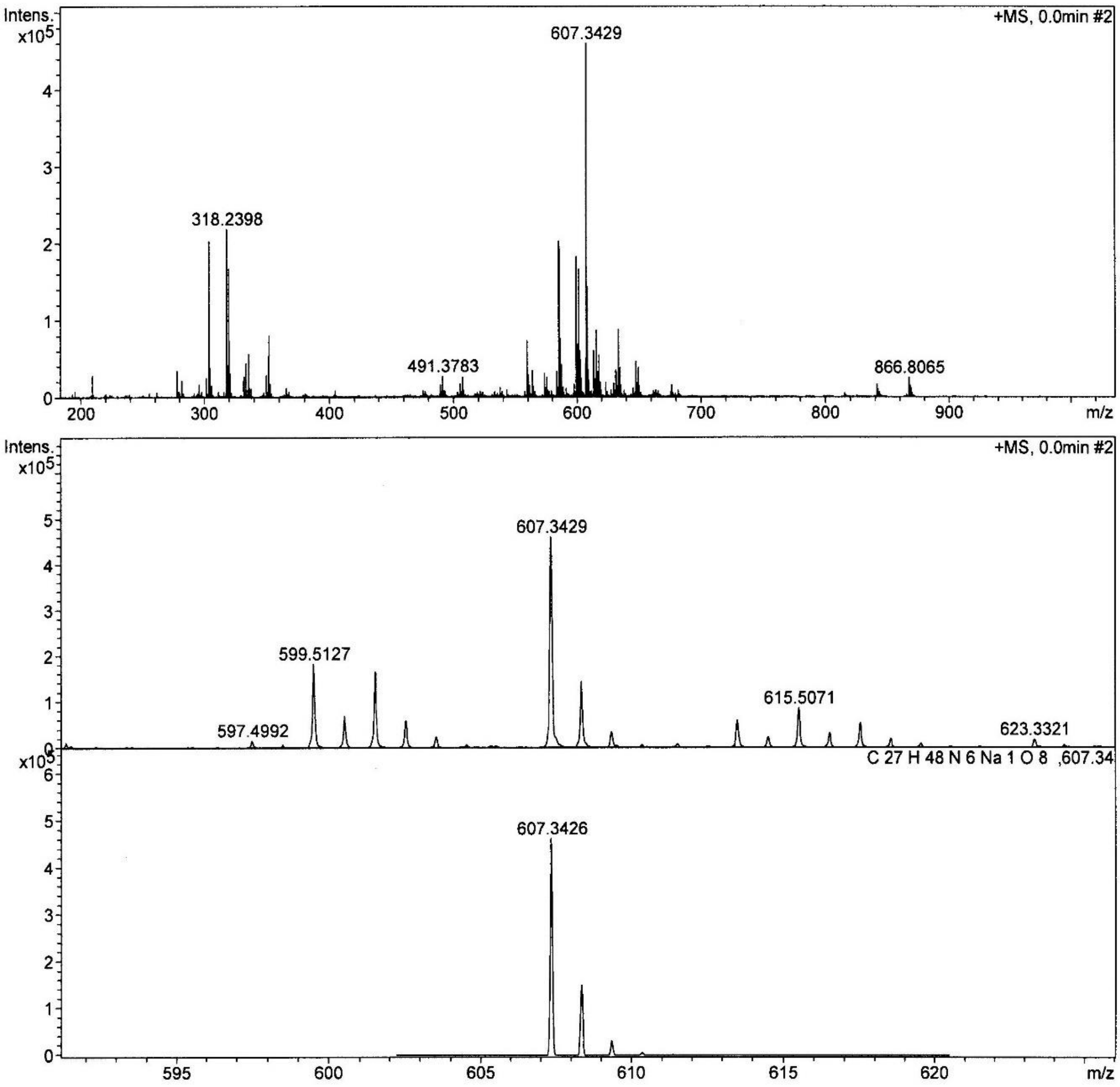

Supplement: Supplementary file 1 [file antibiotics-12-01467-s001.zip › antibiotics-2548788-supplementary.pdf]
